# Electron flow in hydrogenotrophic methanogens under nickel limitation

**DOI:** 10.1038/s41586-025-09229-y

**Published:** 2025-07-02

**Authors:** Shunsuke Nomura, Pablo San Segundo-Acosta, Evgenii Protasov, Masanori Kaneko, Jörg Kahnt, Bonnie J. Murphy, Seigo Shima

**Affiliations:** 1 Microbial Protein Structure Group, Max Planck Institute for Terrestrial Microbiology, Marburg, Germany; 2 Redox and Metalloprotein Research Group, Max Planck Institute of Biophysics, Frankfurt am Main, Germany; 3 Structural Biology Programme, Spanish National Cancer Research Centre (CNIO), Madrid, Spain; 4 Geological Survey of Japan, National Institute of Advanced Industrial Science and Technology (AIST), Tsukuba, Japan; 5 Core Facility for Mass Spectrometry & Proteomics, Max Planck Institute for Terrestrial Microbiology, Marburg, Germany

**Keywords:** Multienzyme complexes, Archaeal physiology, Proteomics, Carbon cycle, Cryoelectron microscopy

## Abstract

Methanogenic archaea are the main producers of the potent greenhouse gas methane^1,2^. In the methanogenic pathway from CO_2_ and H_2_ studied under laboratory conditions, low-potential electrons for CO_2_ reduction are generated by a flavin-based electron-bifurcation reaction catalysed by heterodisulfide reductase (Hdr) complexed with the associated [NiFe]-hydrogenase (Mvh)^3–5^. F_420_-reducing [NiFe]-hydrogenase (Frh) provides electrons to the methanogenic pathway through the electron carrier F_420_ (ref. ^6^). Here we report that under strictly nickel-limited conditions, in which the nickel concentration is similar to those often observed in natural habitats^7–11^, the production of both [NiFe]-hydrogenases in *Methanothermobacter marburgensis* is strongly downregulated. The Frh reaction is substituted by a coupled reaction with [Fe]-hydrogenase (Hmd), and the role of Mvh is taken over by F_420_-dependent electron-donating proteins (Elp). Thus, Hmd provides all electrons for the reducing metabolism under these nickel-limited conditions. Biochemical and structural characterization of Elp–Hdr complexes confirms the electronic interaction between Elp and Hdr. The conservation of the genes encoding Elp and Hmd in CO_2_-reducing hydrogenotrophic methanogens suggests that the Hmd system is an alternative pathway for electron flow in CO_2_-reducing hydrogenotrophic methanogens under nickel-limited conditions.

## Abstract (web summary)

Two distantly related methanogens shift the methanogenic pathway under nickel limitation, suggesting that methanogens adapt to changing bioavailability of nickel by rerouting electron flow between two different pathways.

## Main

Methanogenic archaea have a substantial effect on global climate through the production of nearly all biogenic methane^1,2^. Understanding the biological reactions driving methane release is essential to devising climate change mitigation strategies. Methanogenic metabolism is typically studied by culturing isolated methanogens in the laboratory and analysing their gene expression, enzymes and metabolites^2^, on the general assumption that this gives an accurate understanding of metabolism in natural environments. However, important differences exist between standard laboratory conditions and natural environments. For example, the concentration of nickel ions in many natural environments is in the nanomolar range^7–11^, but the standard synthetic medium for methanogens contains more than 100 times higher concentrations for better growth rate and yield^12^. This is because several key enzymes in the hydrogenotrophic methanogenic pathway, namely, [NiFe]-hydrogenases for oxidation of H_2_ and methyl-coenzyme M reductase (Mcr) for production of methane, contain a nickel cofactor as the prosthetic group^13,14^. In addition, the enzymes involved in anabolic CO_2_ fixation, carbon monoxide dehydrogenase and acetyl-coenzyme A synthase, also contain nickel within the active-site cofactor^15^.

The canonical CO_2_-reducing hydrogenotrophic methanogenic pathway contains three types of [NiFe]-hydrogenase^7,16^, namely, F_420_-reducing [NiFe]-hydrogenase (Frh), heterodisulfide reductase (Hdr)-associated [NiFe]-hydrogenase (Mvh) and membrane-associated energy-converting [NiFe]-hydrogenases (Eha and Ehb). Frh provides electrons from H_2_ to the soluble electron carrier F_420_ to produce the reduced form, F_420_H_2_ (ref. ^6^). F_420_H_2_ is used for two reduction steps within the methanogenic pathway^2,16^. Low-potential electrons required for the spatially coupled reduction and fixation of CO_2_ are generated by a flavin-based electron-bifurcation (FBEB) reaction catalysed by an enzyme complex of Hdr. The Mvh–Hdr complex catalyses bifurcation of electrons from H_2_ (refs. ^4,5^), thereby coupling the low-potential CO_2_ reduction and fixation^4^ with a high-potential reduction of the heterodisulfide of coenzyme M and coenzyme B (CoM-S-S-CoB)^3^. The Hdr complexes form a stable megacomplex with formyl-methanofuran dehydrogenase isoenzymes (Fmd or Fwd), which are two isoenzymes containing molybdenum or tungsten as part of the metallocofactor, molybdopterin or tungstopterin, respectively^17–19^. Low-potential electrons that are used for the biosynthetic reactions in the cell are replenished by Eha and Ehb, which oxidize H_2_ to produce low-potential electrons powered by dissipation of the membrane potential^20^.

It has been reported that in medium containing a low concentration of nickel ions (200 nM Ni^2+^), the production of Frh is strictly downregulated and its function is substituted by a nickel-free enzyme system composed of [Fe]-hydrogenase, which is also known as H_2_-forming methylene-tetrahydromethanopterin (methylene-H_4_MPT) dehydrogenase (Hmd), and F_420_-dependent methylene-H_4_MPT dehydrogenase (Mtd)^21^. Hmd catalyses reversible heterolytic cleavage of H_2_ and hydride transfer to methenyl-H_4_MPT^+^ to form methylene-H_4_MPT. Mtd catalyses reversible hydride transfer from methylene-H_4_MPT to F_420_ to form F_420_H_2_. Therefore, the coupled Hmd+Mtd reaction catalyses H_2_-dependent reduction of F_420_ to F_420_H_2_, which is the same reaction catalysed by Frh^21,22^ (Supplementary Fig. 1).

Here we report that under nickel-limiting conditions, in *Methanothermobacter marburgensis*, F_420_-dependent electron-donating proteins (Elp) are produced and form a complex with Hdr. The Elp–Hdr complex uses F_420_H_2_ for the Hdr reaction, fully substituting Mvh as an electron donor module for FBEB at lower nickel concentrations (<50 nM), at which Mvh production is not detected. Under these conditions, no [NiFe]-hydrogenases are used in the main methanogenesis pathway, and all eight electrons for reduction of CO_2_ to methane are supplied via F_420_H_2_ regenerated by the Hmd+Mtd system. The Elp–Hdr complex forms a supercomplex with Fmd or Fwd to perform electron-bifurcating CO_2_ reduction using F_420_H_2_ as an electron donor. Cryogenic electron microscopy (cryo-EM) structures of the Elp–Hdr complex show that Elp interacts with and can transfer electrons to Hdr. The wide co-distribution of the genes encoding Elp and Hmd in methanogens and expression of the corresponding electron transfer pathways in *M.* *marburgensis* and in the distantly related species *Methanothermococcus thermolithotrophicus* indicates that methanogenesis using the Elp–Hdr complex and Hmd contributes to the survival of CO_2_-reducing hydrogenotrophic methanogenic archaea under nickel-limited conditions.

## Proteome under nutrient limitation

**Fig. 1:**
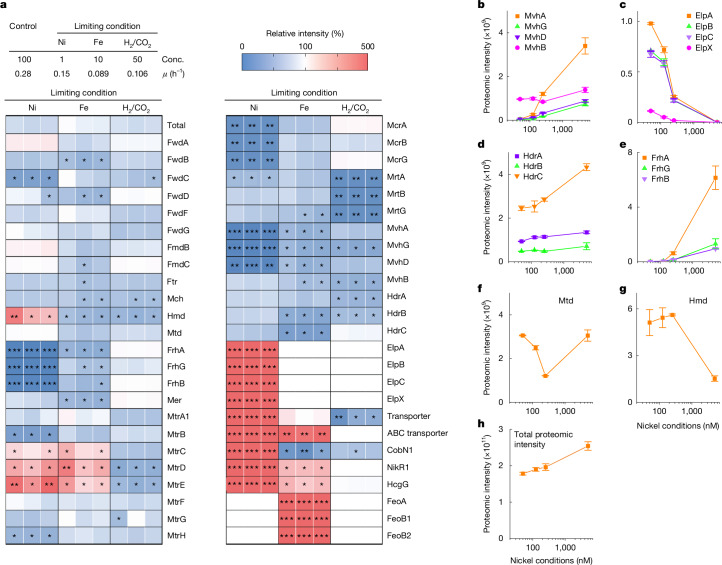
Proteomic analysis of *M.marburgensis* cells under limiting conditions. **a**, The table at the top shows the reduction rate (%) of the Ni^2+^ and Fe^2+^ concentrations, and the flow rate of H_2_/CO_2_ gas in the limitation culture (Conc.), and the specific growth rate (*μ* (h^−1^)) of the cultures used for proteomic analysis. Ni^2+^ and Fe^2+^ in the standard condition are 5 μM and 50 μM, respectively. The total intensity of the mass spectrometry-based proteomic analysis (Total) and the intensity of the individual proteins of three distinct samples are calculated as a percentage of the values obtained under the standard culture condition and the relative intensities are shown by a heat map. Proteins related to the CO_2_-reducing hydrogenotrophic methanogenic pathway are shown. Ftr, formyltransferase; Mch, cyclohydrolase; Mer, methylene-H_4_MPT reductase; Transporter, MTBMA_c10530; ABC transporter, MTBMA_c10830; Feo, ferrous iron transport protein. *15–50% or 200–400%; **5–15% or 400–1,000%; ***<5% or >1,000%. **b**–**h**, The proteomic intensity of MvhA, MvhB, MvhD, MvhG (**b**), ElpA, ElpB, ElpC, ElpX (**c**), HdrA, HdrB, HdrC (**d**), FrhA, FrhB, FrhG (**e**), Mtd (**f**), Hmd (**g**) and total (**h**) from samples from the cells cultivated under various nickel conditions (5,000 nM, 250 nM, 125 nM and 50 nM). Means of the proteome intensity of three distinct samples are shown and error bars indicate standard error (s.e.). Source data

**Extended Data Fig. 1:**
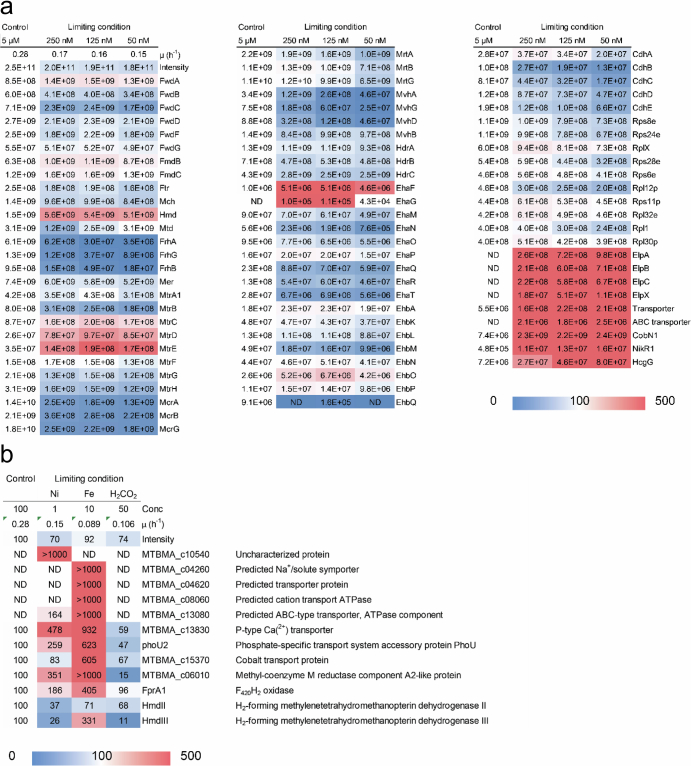
Proteomic analysis of *Methanothermobacter marburgensis* cells under limiting conditions. **a**. Change of the expression under 250 nM, 125 nM, and 50 nM Ni^2+^ concentrations. The control culture contained 5 µM Ni^2+^. Means of the proteome intensity of distinct samples (n = 3) are shown and error bars indicate standard error (SE). Membrane-associated [NiFe]-hydrogenases (Eha and Ehb). CO dehydrogenase (Cdh), 30S ribosomal protein (Rps), 50S ribosomal protein (Rpl). For other abbreviations, see Fig. 1 of the main text. The up-regulation of production of “Transporter” and “ABC transporter” suggests that these proteins could be transporters involved in Ni transport. **b**. In this figure, proteins that are not involved in the CO_2_-reducing hydrogenotrophic methanogenic pathway but exhibited changes in protein intensity are shown. The concentration (Conc) of Ni^2+^ and Fe^2+^, the total intensity of the mass spectrometry-based proteomic analysis (Intensity) and the proteome intensity of the individual proteins are shown as a percentage of the values obtained under the standard culture condition (Control), in which the concentration of Ni^2+^ and Fe^2+^ in the control are 5 μM and 50 μM, respectively. Means of three distinct samples are shown. The specific growth rate (μ) was calculated from the dilution rate of the continuous flow rate of the cultures used for the proteomic analysis. The product of the genes up-regulated under the Ni^2+^ and Fe^2+^-limited condition might be involved in transport of the respective metal ion. Source data

To better understand the metabolism of CO_2_-reducing hydrogenotrophic methanogens in natural environments, we cultivated *M.* *marburgensis* under Ni^2+^-, Fe^2+^- and substrate (H_2_ + CO_2_)-limited conditions in a continuous-flow culture (Supplementary Fig. 2). The cells were collected, and the protein profile was determined by proteomic analysis, in which proteolytically processed cellular proteins were analysed by mass spectrometry (Fig. 1). The intensity of the total proteins and ribosomal proteins (Fig. 1h and Extended Data Fig. 1a) in the proteomic data slightly decreased to about 70% under strong nutrient limitation, even though equal amounts of proteins or peptides were analysed for all conditions. Therefore, the change of intensity of individual proteins should be assessed in the context of the general decrease in the intensity of the proteomic data.

Under Ni^2+^- and Fe^2+^-limited conditions, the production of the corresponding transporter increased, suggesting that limitation of the ions in the medium is partially compensated by higher capacity for ion import (Fig. 1a and Extended Data Fig. 1). In the Fe^2+^-limited condition, the levels of methanogenic enzymes were not significantly changed, except for a twofold to fourfold increase in the three membrane-integrated subunits MtrCDE of energy-conserving methyltransferase (MtrA−H). MtrA−H catalyses exergonic methyl transfer from methyl-H_4_MPT to coenzyme M^23^, which is coupled with energy-conserving sodium ion translocation. The *mtrCDE* genes of *M.* *marburgensis* are contained within the *mtrA−H* gene cluster^24^. A similar increase of the MtrCDE proteins was observed in the Ni^2+^-limited condition but not in the H_2_ + CO_2_-limited condition. Under H_2_ + CO_2_ limitation, no significant change was observed for known metabolic enzymes except for a decrease in isoenzyme II of methyl-coenzyme M reductase (Mrt), as described previously^25^.

In the Ni^2+^-limited culture, significant changes in the production of the methanogenic enzymes were detected, especially for nickel-containing enzymes. The production of Mcr decreased to about 10% of the standard culture condition. The production of the isoenzyme Mrt also decreased, but to a lesser extent (about 50%; Fig. 1a and Extended Data Fig. 1a), resulting in a change in the ratio of Mcr isoenzymes. CO dehydrogenase and Eha and Ehb may be downregulated, although the interpretation is difficult owing to the variable effect on the individual subunits (Extended Data Fig. 1a). Possible metal-chelating proteins, a hypothetical cobalamin biosynthesis protein annotated as CobN1, and a homologue of nickel-responsive transcriptional regulator NikR1 are produced at high levels only under nickel limitation. As reported previously^21,26^, two cytosolic [NiFe]-hydrogenases (FrhAGB and MvhAGD) decreased markedly under Ni^2+^ limitation, disappearing almost entirely at 125 nM Ni^2+^ (FrhAGB) and 50 nM Ni^2+^ (MvhAGD). In contrast to the MvhAGD proteins, one protein encoded in the *mvh* gene cluster, MvhB (ref. ^27^), was constitutively expressed. MvhB is a polyferredoxin of unknown function containing 12 [4Fe–4S] clusters^27–29^. Recently, MvhB has been isolated in the Mvh–Hdr–Fmd (or Fwd) complex from *M.* *marburgensis*^19^. A previous study indicated that transcription of the *mvhAGDB* operon was not downregulated under low nickel concentrations^26^.

**Fig. 2:**
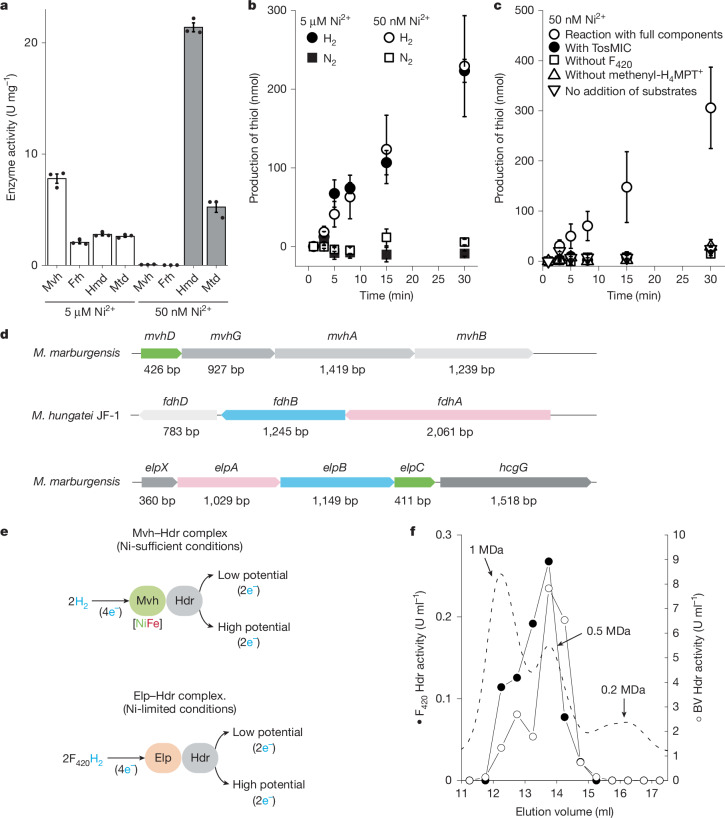
Enzymological analysis of the Hdr complex from *M. marburgensis* cells and purification of the Elp–Hdr complex. **a**–**c**, Activity of the enzymes in the cell extract. Means of three measurements of distinct samples obtained from the same cell extract (*n* = 1) are shown and error bars indicate s.e. **a**, The enzyme activity under nickel-sufficient and nickel-limiting conditions. **b**, H_2_-dependent Hdr activity is shown by production of thiols (CoM-SH and CoB-SH) from CoM-S-S-CoB using cell extract. The cell extracts from the nickel-sufficient culture (5 µM Ni^2+^; filled label) and nickel-limiting culture (50 nM Ni^2+^; open label) were tested under either H_2_ or N_2_. **c**, H_2_-dependent Hdr activity of the washed cell extracts from the nickel-limiting cultured cells was tested. To confirm the activity, addition of the Hmd-specific inhibitor toluenesulfonylmethyl isocyanide (TosMIC) and the conditions containing only one substrate (without F_420_ or without methenyl-H_4_MPT^+^) or lacking both substrates (no addition of substrates) were tested. **d**, Gene clusters encoding the proteins associated with Hdr. bp, base pairs. **e**, FBEB reaction catalysed by the Mvh–Hdr complex from *M.* *marburgensis* under nickel-sufficient conditions and the Elp–Hdr complex from *M.* *marburgensis* under nickel-limiting conditions. **f**, Characterization of the fractions eluted from size-exclusion chromatography of the Hdr complex from *M.* *marburgensis* cells from nickel-limiting continuous culture (50 nM Ni^2+^). Hmd+Mtd-coupled-reaction-mediated F_420_-dependent Hdr (F_420_ Hdr) activity and benzyl-viologen-dependent Hdr (BV Hdr) activity are shown. Absorbance at 280 nm (arbitrary units) is shown as a dashed line. Source data

Levels of Hmd increased under Ni^2+^ limitation, consistent with the fact that the Frh reaction can be replaced by the coupled Hmd+Mtd reactions. Accordingly, the production of the enzymes involved in the biosynthesis of the prosthetic group of Hmd (HcgG) increased significantly under nickel limitation (Extended Data Fig. 1a). By contrast, the proteomic data did not show a monotonic increase in Mtd as described by a previous enzyme assay-based study^21^. Our proteomic data show a decrease in Mtd with decreasing Ni^2+^ concentrations from 5 μM to 250 nM followed by an increase in Mtd levels as Ni^2+^ concentrations fall below 250 nM (Fig. 1f). Our enzyme assays indicate an approximately twofold increase in the Mtd activity in the 50 nM Ni^2+^ condition compared to the standard culture condition (Fig. 2a).

## Assay of H_2_-dependent Hdr activity

Continuous-culture experiments in this study showed that *M.* *marburgensis* grows relatively well even at 50 nM Ni^2+^, with specific growth rates reduced by around half compared to those in nickel-sufficient conditions. In the 50 nM Ni^2+^ condition, the production of Hdr did not significantly decrease when taking into account the negative effect on total proteome intensity caused by nickel limitation (Fig. 1d). These data indicate that H_2_-dependent Hdr activity should be largely maintained in cells under nickel limitation. This is confirmed by enzyme assays indicating that the cell extract from *M.* *marburgensis* cultivated under 50 nM Ni^2+^ catalyses H_2_-dependent Hdr activity at a similar rate to a standard culture with 5 μM Ni^2+^ (Fig. 2b). As the only cytosolic hydrogenase in the strictly nickel-limited cell is Hmd, it is likely that the Hmd+Mtd system supplies electrons to the H_2_-dependent Hdr reaction. To test this hypothesis, we prepared a washed cell extract, from which the small molecules were depleted by successive rounds of ultrafiltration and dilution. An assay of the H_2_-dependent Hdr activity using the washed cell extract from the 50 nM Ni^2+^ culture showed activity that was dependent on externally added methenyl-H_4_MPT^+^ and F_420,_ and was inhibited by toluenesulfonylmethyl isocyanide, a specific inhibitor of Hmd^30,31^ (Fig. 2c). These results indicate that, under strict nickel limitation, electrons for H_2_-dependent Hdr activity are provided by the Hmd+Mtd system, with the direct electron donor to the Hdr reaction probably being F_420_H_2_. In this case, we expect Hdr in the nickel-limited cells to form a complex with a protein module that can accept electrons from F_420_H_2_.

Previously, we showed that a complex of formate dehydrogenase (FdhAB–MvhD) and HdrABC can catalyse F_420_H_2_-dependent FBEB, probably through an FAD-containing active site in the FdhB subunit^18^, which is homologous to the F_420_-reducing subunit of the soluble Frh complex^6^. Although the cell extract of *M.* *marburgensis* did not exhibit Fdh activity under either nickel-sufficient or nickel-limiting conditions, our proteomic data do indicate upregulation of proteins exhibiting homology to FdhAB (Fig. 1c). On the basis of homology and proteomic data, we selected MTBMA_c15240, MTBMA_c15230, MTBMA_c15220 and MTBMA_c15210, which are encoded in a gene cluster, for further study. We designate the products of these genes ElpX, ElpA, ElpB and ElpC (Fig. 2d,e), respectively. As Ni^2+^ concentrations are lowered, the level of Elp proteins increases in proportion to the decrease in the Mvh subunits, and the maximum proteomic intensity across different Ni^2+^ conditions is similar for Elp and Mvh (Fig. 1b,c). ElpA, ElpB and ElpC are homologues of the Hdr-associated Fdh subunits, FdhA and FdhB, and a subunit of Mvh (MvhD), respectively^18^ (Fig. 2d). Whereas FdhA contains a molybdopterin cofactor as the prosthetic group for oxidation of formate, its homologue ElpA lacks the region for molybdopterin binding, which indicates that ElpA is devoid of Fdh activity. MvhD functions as the electron-transferring connector between Fdh and Hdr^18^ or Mvh and Hdr^5^ in previously characterized complexes. We reasoned that ElpABC forms a complex with Hdr to catalyse F_420_H_2_-dependent Hdr activity and substitute the function of the Mvh–Hdr complex (Fig. 2e) under strictly nickel-limited conditions.

## Purification of the Elp–Hdr–Fmd complex

**Extended Data Fig. 2:**
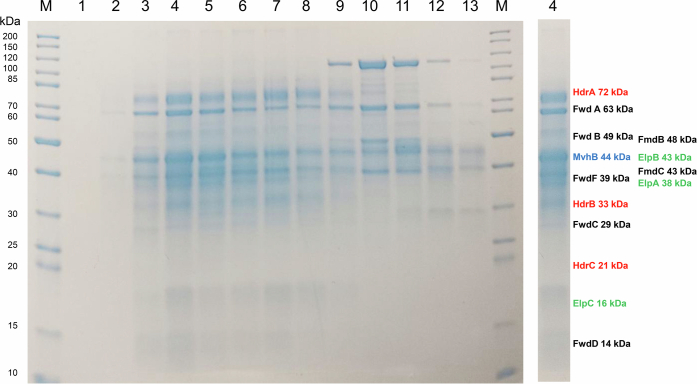
SDS-PAGE of the size exclusion chromatography (Superose 6 Increase) fractions. (Left) Fractions 1–13 every 0.5 ml starting at 11.25 ml, shown in Fig. 2f, were analyzed by SDS-PAGE using 4 -16 % gradient polyacrylamide gel from Bio-Rad Laboratories. The eluted fraction was concentrated 10-fold through a 3-kDa ultrafilter and 10 μl were subjected to denaturation in SDS and loaded onto the gel. (M) Marker proteins with the molecular mass. (Right) The SDS-PAGE lane of fraction 4 containing the 1-MDa complex is shown to indicate the deduced position of the subunits of Elp, Hdr, and Fmd (Fwd). Four experiments were repeated independently with similar results (for the uncropped data for the gel, see Supplementary Fig. 7).

**Extended Data Fig. 3:**
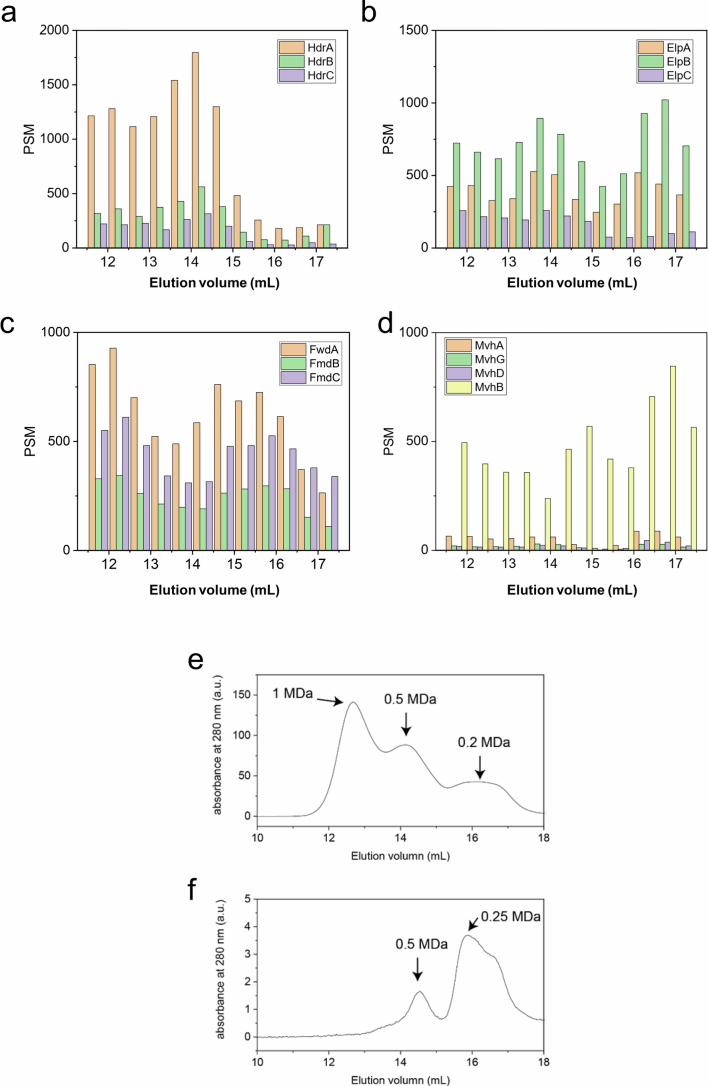
Proteomic analysis of the size-exclusion chromatography step of fractionation of the Elp-Hdr-Fmd complex. **a-d**. Mass spectrometry-based proteomic analysis of the size-exclusion chromatography step of fractionation of the Elp-Hdr-Fmd complex shown in panel Fig. 2f. HdrABC (**a**), ElpABC (**b**), FwdA, FmdB, and FmdC (**c**), and MvhAGDB (**d**). The total number of identified peptide spectra matches for the protein (PSM) (n = 1) is shown as the intensity of the proteins in the ordinate. **e**. Purification from the nickel-limiting cells (50 nM Ni^2+^), from disruption of the cells through the three chromatographic steps, was completed within 1 day. **f**, Re-chromatography of the 1-MDa fraction from panel **e** after 40 h storage on ice. Although stability testing of the Elp-Hdr-Fmd complex of the purified 1-MDa complex was only conducted once, the elution profiles from the multiple purification processes, which differed in terms of the size of the 1-MDa peak, support that the complex is unstable. Source data

To test whether the Elp proteins substitute the Mvh subunits in the nickel-limited cells, we purified the predicted Elp–Hdr complex from cells cultivated in the presence of 50 nM Ni^2+^ by following the Hdr activity using anion-exchange-, hydrophobic-interaction and size-exclusion chromatography. In the fractions of the size-exclusion chromatography, Hdr activity was detected in two symmetric elution peaks at 1 MDa and 0.5 MDa, which were followed by a broad asymmetric peak at 0.2 MDa with no Hdr activity (Fig. 2f and Extended Data Fig. 2). We performed proteomic analysis of the fractions (Extended Data Table 1 and Extended Data Fig. 3a–d). In the 1-MDa and 0.5-MDa fractions, ElpABC, HdrABC, MvhB, FwdABCDFG and FmdBC were detected. In the 0.2-MDa fraction, the main proteins in the last parts were identified as ElpAB. These data indicate that ElpABC probably forms a 1-MDa complex with Hdr and Fmd or Fwd. The apparent molecular mass and the protein composition suggest that the 1-MDa fraction contains the dimeric ElpABC–HdrABC–MvhB–FwdABCDFG supercomplex. The additional presence of FmdBC, and the intensity of the Fmd and Fwd subunits, suggests that in some copies of the supercomplex, the FwdBC proteins are replaced by their molybdenum-dependent isoenzyme subunits. The fractions containing ElpAB showed no Fdh activity. For the sake of clarity, we will refer to the supercomplex as the Elp–Hdr–Fmd complex. Similar megacomplexes of Mvh, Hdr and Fmd (or Fwd), and Fdh, Hdr and Fmd have been isolated from *M.* *marburgensis*^19^ and *Methanospirillum hungatei*^18^, respectively.

Both 1-MDa and 0.5-MDa fractions showed F_420_H_2_-dependent Hdr activity (Fig. 2f), indicating that the 1-MDa and 0.5-MDa fractions contain Hdr complexes with differing subunit compositions. The specific activities were 0.13 (±0.01) U mg^−1^ (*n* = 3) for the 1-MDa fraction and 0.40 (±0.06) U mg^−1^ (*n* = 3) for the 0.5-MDa, which are comparable to the H_2_-dependent Hdr activity of the Mvh–Hdr complex purified from *M.* *marburgensis*^3^. These results demonstrate the presence of a functional Elp–Hdr complex in *M.* *marburgensis* under nickel-limited conditions.

The broad distribution of the subunits of Elp, Hdr, Fmd (or Fwd) and MvhB in the size-exclusion chromatography fractions indicated that the protein complex is unstable and partially dissociated into smaller subcomplexes. To assess the stability of the 1-MDa complex, we reloaded the 1-MDa fraction onto the same size-exclusion chromatography column. The resulting elution profile did not reproduce a single 1-MDa peak, rather, lower molecular weight peaks appeared (Extended Data Fig. 3e,f), confirming the instability of this supercomplex.

## Cryo-EM structure of Elp–Hdr

**Fig. 3:**
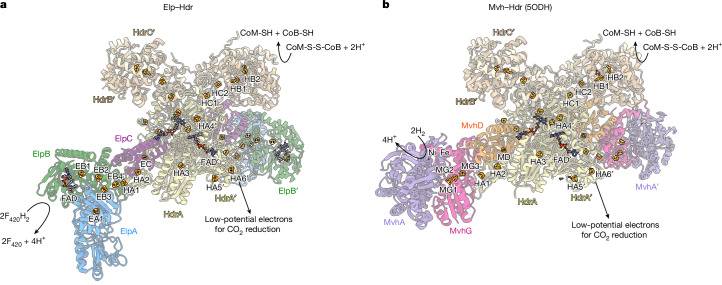
Structure and reaction of the Elp–Hdr complex from *M. marburgensis.* **a**,**b**, In the Elp–Hdr complex (**a**), the ElpABC subunits interact with the Hdr dimer in a manner similar to that observed in the Mvh–Hdr dimer composed of MvhAG, MvhD and HdrABC from *M. thermolithotrophicus* (**b**; Protein Data Bank (PDB) accession code 5ODH). F_420_H_2_ is oxidized at the ElpB FAD-binding site, and electrons are transferred through the cubane [4Fe–4S] clusters of ElpB (EB(1, 2, 4)) and of the N terminus of HdrA ((HA(1,2)) to reach the [2Fe–2S] cluster of ElpC (EC), from which they are presumably transferred to the bifurcating HdrA′ FAD′ by an unknown mechanism (Extended Data Fig. 5c).

**Extended Data Fig. 4:**
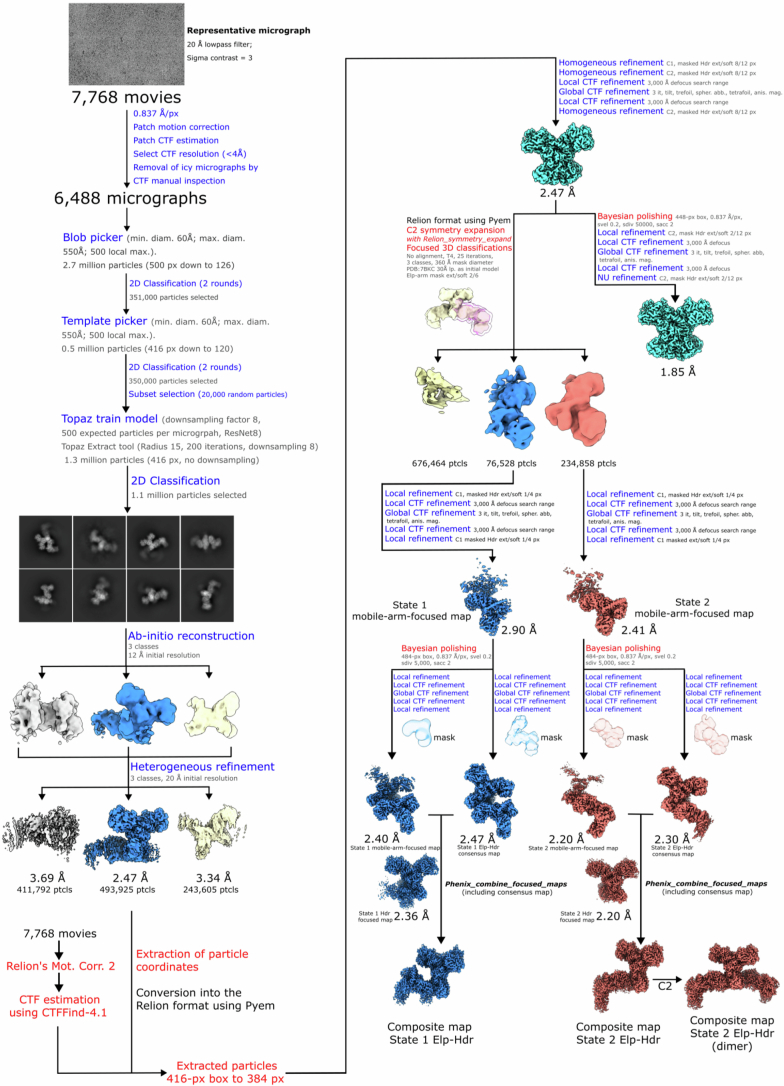
Image processing workflow for the analysis of the Elp-Hdr dataset. State 1 and State 2 correspond to the two different conformational states of the mobile arm composed of ElpA, B and C, and the N- and C-terminal regions of HdrA. Resolutions were estimated using the gold-standard FSC at 0.143. Steps carried out in CryoSPARC are shown in blue, whereas steps carried out in Relion 4.0 are shown in red. Abbreviations used: External (ext.), pixel (px), per (p.), resolution (res.), particles (ptcls), including (incl.), extension (ext.), soft-edge (soft), classification (class.), iterations (it.), excluding (excl.), spherical aberration (spher. abb.), anisotropic magnification (anis. mag.) and B-factor (Bfac.).

**Extended Data Fig. 5:**
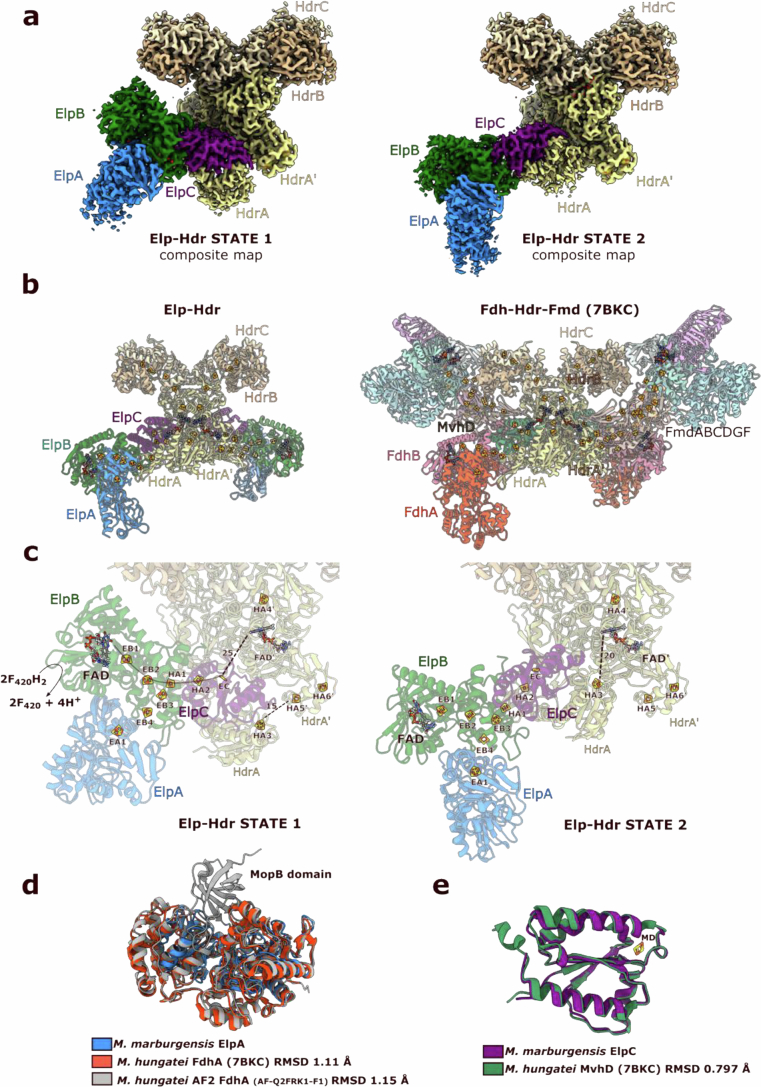
The Elp-Hdr complex of *M. marburgensis* is structurally similar to the Fdh-Hdr region of the Fdh-Hdr-Fmd complex of *M. hungatei.* **a**, Composite maps of the two different conformational states of the Elp-Hdr complex, which are very similar to the states 1 and 2 described for Fdh-Hdr-Fmd in *M. hungatei*^18^. **b**, The structure of the Elp-Hdr complex (left) is highly similar to the Fdh-Hdr region of the Fdh-Hdr-Fmd complex (right) of *M. hungatei* (PDB:7BKC). **c**, Atomic models of the conformational states 1 (left) and 2 (right) of the Elp-Hdr mobile arm composed of subunits ElpA (blue), B (green) and C (purple), and the N- and C-terminal domains of HdrA (khaki). Following the proposed model of electron transfer in Fdh-Hdr-Fmd^18^, in Elp-Hdr State 1 the [2Fe-2S] cluster of ElpC (EC) moves closer to the bifurcating FAD’ of HdrA’ (25 Å distance) to transfer two electrons through an unknown mechanism. The complex transitions to State 2 through the rotation of the mobile arm, by which the [4Fe-4S] cluster of the HdrA C terminus (HA3) moves closer to the reduced FAD’ (20 Å distance). The high-potential electron is transferred from the hydroquinone state of FAD’ to CoM-S-S-CoB via HA4’, whereas the low-potential electron from the flavosemiquinone state is transferred to HA3, the ‘shuttle cluster’. A transition back to conformational state 1 brings HA3 near enough to HA5’ for efficient electron transfer. **d**, ElpA (blue) is highly similar to FdhA (PDB:7BKC, orange), but lacks the molybdopterin-binding (MopB) domain and is inactive for the Fdh reaction. The AlphaFold2 model of *M. hungatei* FdhA (AF-Q2FRK1-F1) is also displayed (grey) to show the MopB domain, which was not deposited for *M. hungatei* FdhA (orange) due to low map resolution^18^; however, unlike for the Elp complex, clear density was observed for the MopB domain of *M. hungatei* FdhA. **e**, ElpC is highly similar to *M. hungatei* MvhD and shows the [2Fe-2S] cluster (EC) for electron transfer to the bifurcating FAD of HdrA.

**Extended Data Fig. 6:**
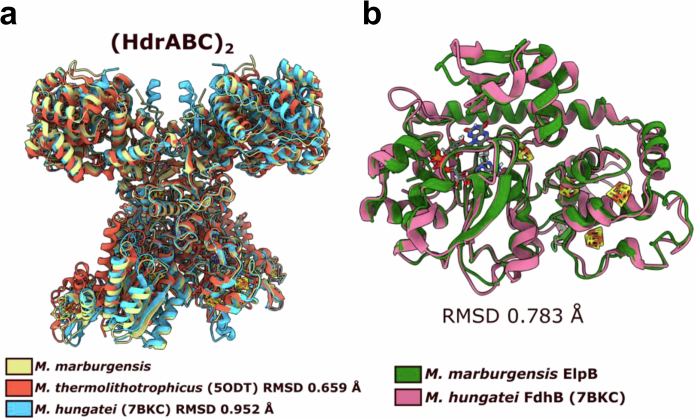
Comparison of the structure of the HdrABC dimer and ElpB from *M. marburgensis* with their homologs. a, The structure of the core (HdrABC)_2_ region in the Elp-Hdr complex is highly similar to the (HdrABC)_2_ of *M. hungatei* (Fdh-Hdr-Fmd complex, PDB:7BKC, root-mean-square deviation (RMSD) of 0.952 Å between 516 amino acids) and the (HdrABC)_2_ of *M. thermolithotrophicus* (Mvh-Hdr complex, PDB:5ODH, RMSD of 0.659 Å between 390 amino acids). b, The ElpB subunit of the Elp-Hdr complex is homologous to the FdhB subunit of the FdhAB-MvhD arm of *M. hungatei* (PDB:7BKC, RMSD 0.783 Å between 328 amino acids).

To further characterize the Elp–Hdr complex, we performed cryo-EM analysis of the 1-MDa fraction (Fig. 3, Extended Data Figs. 4–6 and Supplementary Figs. 3–6). We obtained structures of the ElpABC–HdrABC complex at resolutions reaching 2.2 Å (Extended Data Table 2, Extended Data Figs. 4 and 5 and Supplementary Figs. 3–6). The complex is a dimer, (ElpABC–HdrABC)_2_, of heterohexamers (Fig. 3). The central (HdrABC)_2_ unit closely resembles those observed previously in Mvh–Hdr^5^ and Fdh–Hdr–Fmd^18^ assemblies (Extended Data Figs. 5 and 6). Focused three-dimensional classification revealed different conformational states of the mobile arm consisting of ElpABC, together with the amino- and carboxy-terminal domains of HdrA, similar to those previously observed for the Fdh–Hdr–Fmd complex of *M.* *hungatei*^18^ (Extended Data Fig. 5a,c). This conformational change seems to be involved in conformational gating of electron transfer for the electron-bifurcation reaction. The architecture of the electron-donor arm, containing ElpABC, is similar to that observed for homologous subunits of the FdhAB–MvhD within the Fdh–Hdr–Fmd supercomplex^18^ (Extended Data Fig. 5b). As predicted from the sequence, ElpA is a truncated form of FdhA and does not bind a molybdopterin cofactor (Extended Data Fig. 5d). The overall structure of ElpB in the cryo-EM structure superposes well with FdhB of the Fdh–Hdr–Fmd complex from *M.* *hungatei*^18^ (Extended Data Fig. 6), in which the positions of FAD and the four [4Fe–4S] clusters are conserved. Structural conservation with related enzymes^5,6,18,32^ allows a confident assessment of the function of the modules. In ElpB, a hydride is transferred from F_420_H_2_ to FAD, and electrons are then transferred from the FAD down a chain of iron–sulfur clusters, eventually reaching the bifurcating FAD in HdrA. ElpC has an identical role to its homologue MvhD in structures of Mvh–Hdr and Fdh–Hdr–Fmd complexes. This subunit binds a conserved [2Fe–2S] cluster near to the bifurcating FAD^5,18^ (Fig. 3 and Extended Data Fig. 5c,e). Cofactors including the electron-bifurcating FAD of HdrA and the CoM-S-S-CoB-reducing non-cubane [4Fe–4S] clusters of HdrB show conserved architecture and coordination (Supplementary Figs. 5 and 6). These conserved structural features strongly indicate that Elp subunits functionally replace Mvh subunits under Ni^2+^ limitation by providing electrons from F_420_H_2_ for the FBEB reaction that drives reduction of both CO_2_ and CoM-S-S-CoB.

**Extended Data Fig. 7:**
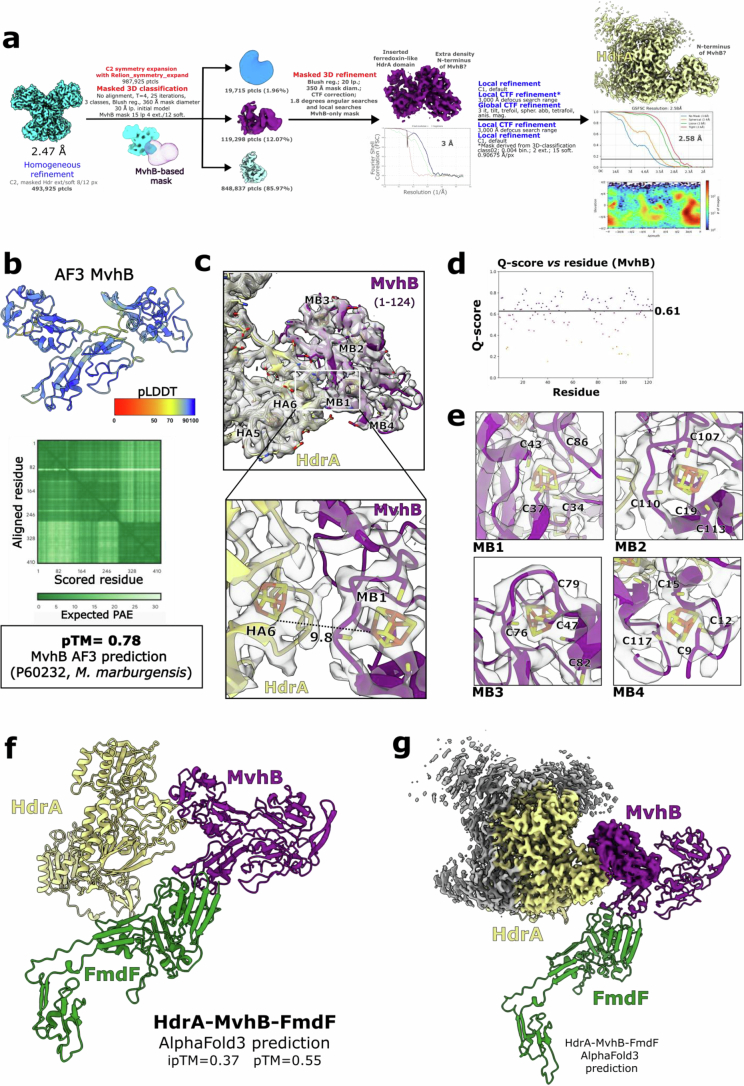
MvhB interacts with the inserted ferredoxin-like domain of HdrA. **a**, 3D classification without alignment masking the AF3-predicted MvhB subunit revealed around 12.1 % of the C2-expanded particles show an additional density bound to the inserted ferredoxin-like domain of HdrA. The map could be refined to 2.58 Å. Processing steps performed in CryoSPARC are shown in blue, whereas steps performed in RELION are shown in red. **b**, AF3-predicted structure of *M. marburgensis* MvhB. The structure is colored according to the per-atom predicted local distance difference test (pLDDT) scores. Predicted alignment error (PAE) scores are also shown. **c**, AF3-predicted MvhB (N-terminal residues 1-57, 67-124) can be fitted into the refined map. The [4Fe-4S] clusters HA6 and MB3 connect HdrA and MvhB electronically (9.8 Å distance). **d**, The AF3-predicted N-terminal region of MvhB displays a mean Q-score of 0.61 when fitted into the map. e, Detail of the densities and fitted [4Fe-4S] clusters (MB1 to MB4) present in the resolved N-terminal region of MvhB. Coordinating cysteines are shown as sticks. **f**, The AF3 structure of HdrA-MvhB-FmdF predicts that MvhB mediates the interaction between Elp-Hdr and Fmd. **g**, The AF3-predicted HdrA (khaki) and MvhB (purple) subunits can be correctly fitted into the HdrA-MvhB map. Other abbreviations used: binarization (bin.), external (ext.), pixel (px), low-pass filtered (lp.), blush regularization (blush reg.), particles (ptcls), extension (ext.), soft-edge (soft), predicted template modeling AF3 score (pTM), interface predicted template modeling AF3 score (ipTM).

In *M.* *marburgensis*, the *mvh* operon contains the *mvhB* gene (Fig. 2d), which encodes the polyferredoxin MvhB^29^. Proteomics analysis of the 1-MDa peak indicates the presence of MvhB as a potential subunit of an Elp–Hdr–Fmd supercomplex. Moreover, a recently isolated potential Mvh–Hdr–Fmd supercomplex also contains MvhB^19^. However, despite repeated attempts, no structure of the Elp–Hdr–Fmd supercomplex could be obtained, probably owing to the instability of this higher-order complex (Extended Data Fig. 3e,f). To assess whether the polyferredoxins MvhB or FmdF could be attached to HdrA, we performed three-dimensional classification without alignment using a mask at the inserted ferredoxin-like domain of HdrA (Extended Data Fig. 7a), which is the binding site of FmdF, and the anticipated point of electron exit from HdrA, in the hexameric Fdh–Hdr–Fmd supercomplex of *M.* *hungatei*^18^. We could observe that about 12% of the particles exhibited additional density. The N-terminal amino acids of MvhB (1–57, 67–124) of the AlphaFold3 (AF3)^33^-predicted structure could be directly fitted in the map with a *Q*-score of 0.61 (Extended Data Fig. 7b–d), and four [4Fe–4S] clusters (here named MB1–MB4) could be modelled at the predicted [4Fe–4S]-binding sites (Extended Data Fig. 7e). The HdrA-bound [4Fe–4S] cluster HA6 is located approximately 9.8 Å from MvhB MB1 (Extended Data Fig. 7c), connecting both subunits electronically. We could also predict the structure of an HdrA–MvhB–FmdF subcomplex using AF3 (Extended Data Fig. 7f,g). Whereas in the *M.* *hungatei* Fdh–Hdr–Fmd supercomplex, the FmdF subunit interacts directly with HdrA^18^, for *M.* *marburgensis*, AF3 predicts that MvhB interconnects HdrA with the Fmd complex via FmdF, so that no direct interaction between Fmd and Elp–Hdr occurs (Extended Data Fig. 7f). Considering that the unresolved MvhB domains are predicted to interact with FmdF, this flexibility could have been caused by the loss of the MvhB–FmdF interaction. These data support the existence of an Elp–Hdr–Fmd supercomplex in the 1-MDa fractions that disassembles after purification.

## Wide co-distribution of Elp and Hmd

**Extended Data Fig. 8:**
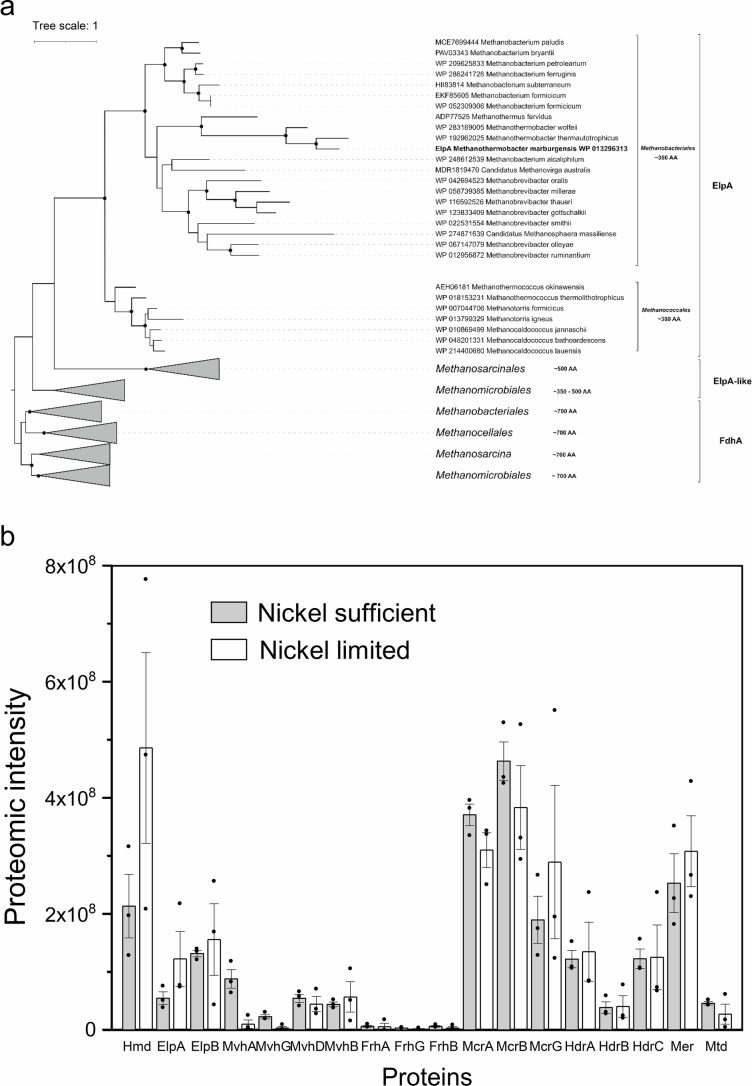
Phylogenetic tree of ElpA and its homologs and the proteomic intensity of samples from cells of *Methanothermococcus thermolithotrophicus.* **a**. Phylogenetic tree of ElpA and its homologs, ElpA-like and FdhA. ElpA, ElpA-like, and FdhA can be separated based on phylogeny and the sequence length (AA, amino-acid sequence length). AlphaFold prediction indicated that the tertiary structure of ElpA-like proteins of Class II methanogens (*Methanosarcinales* and *Methanomicrobiales*) shows higher similarity to that of FdhA. Exceptionally, the tertiary structure of ElpA-like from *Methanocorpusculum* species is structurally almost identical to ElpA from Class I methanogens (*Methanobacteriales* and *Methanococcales*) (see also Supplementary Table 1). The maximum-likelihood tree is based on a MUSCLE alignment and was generated using IQ-TREE with LG + I + G4 model of evolution. Node support was tested with nonparametric bootstrap analysis (filled circles indicate support 70% or higher; 100 replicates). The scale bar indicates the number of substitutions per site. The tree was rooted with various bacterial FdhA. **b**. The proteomic intensity from *M. thermolithotrophicus* (DSM 2095) cultivated under nickel-sufficient (5 μM Ni^2+^) and nickel-limited (50 nM) conditions. Means of the proteome intensity (iBAQ) of the three distinct samples are shown and error bars indicate standard error (SE) (n = 3). Hmd, WP_018153721.1; ElpA, WP_018153231.1; ElpB, WP_018153230.1; MvhA, WP_018154262.1; MvhG, WP_018154261.1; MvhD, WP_018154260.1; MvhB, WP_018154263.1; FrhA, WP_018154259.1; FrhG, WP_018154257.1; FrhB, WP_018153424.1; McrA, WP_018153522.1; McrB, WP_018153526.1; McrG, WP_018153523.1; HdrA, WP_018154264.1; HdrB, WP_018154154.1; HdrC, WP_018154153.1; Mer, WP_026182932.1; Mtd, WP_018154202.1. Proteome intensity of the subunits of the isoenzymes of MvhAGDB encoded in the VhuAGDB gene cluster (WP_018154225.1, WP_018154224.1, WP_245547903.1, and WP_018154226.1) was very low. Isoenzymes of HdrBC (WP_018154154.1 and WP_018154031.1) were not detected. Proteome intensity of McrABG (WP_018154763.1, WP_018154760.1, and WP_018154762.1) was much lower than the counterpart. Therefore, proteome intensity of these isoenzymes is not shown in this figure. Source data

Comparative genomic analysis indicated that the gene cluster encoding ElpAB is widely distributed in the genomes of CO_2_-reducing hydrogenotrophic methanogenic archaea (Extended Data Fig. 8a and Supplementary Table 1). Methanogens harbouring Hmd typically also possess the genes encoding ElpAB. Most genomes containing Hmd but lacking Elp encode FdhA and FdhB. In such organisms, the F_420_H_2_-oxidizing site of FdhB should allow an Fdh–Hdr complex^18^ to substitute the function of Elp under Ni^2+^ limitation. These findings suggest that the nickel-dependent transition of the electron-donating pathway observed in *M.* *marburgensis* may be a broadly conserved feature of CO_2_-reducing hydrogenotrophic methanogens containing Hmd.

**Fig. 4:**
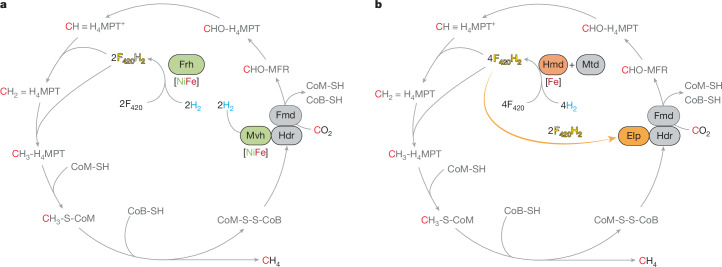
Transition of the hydrogenase and electron-donating system in methanogenesis from CO_2_ and H_2_ under strictly nickel-limiting conditions. **a**,**b**, Schematic views of the methanogenic pathway under nickel-sufficient (**a**) and nickel-limiting (**b**) conditions. Two [NiFe]-hydrogenases, Frh and Mvh, are strongly downregulated under the strictly nickel-limiting conditions. Their functions are substituted by the coupled enzyme system of [Fe]-hydrogenase (Hmd) with Mtd, and Elp complexed with Hdr. The drawing of Hmd and Mtd in **b** indicates a coupled reaction rather than a complex formation. Figure adapted from ref. ^19^ under a Creative Commons licence CC BY 4.0.

To test whether our findings on the nickel-independent pathway can be generalized to other hydrogenotrophic methanogens, we performed a proteomic analysis of *M.* *thermolithotrophicus*, which is phylogenetically distantly related to *M.* *marburgensis* and belongs to the Methanococcales order. Proteomic analysis was performed for samples obtained from cells grown under nickel-sufficient (Ni^2+^ = 5 μM) and nickel-limited (Ni^2+^ = 50 nM) conditions (Extended Data Fig. 8b). In *M.* *thermolithotrophicus*, ElpAB were detected under the nickel-sufficient condition and their production was slightly increased under the nickel-limited condition. The proteome intensity of FrhAGB was much lower than that of Hmd and was not changed by the nickel concentration, which indicates that Hmd+Mtd always functions as the main F_420_-reducing system in this methanogen. The Hdr-associated [NiFe]-hydrogenase MvhAG was significantly decreased under the nickel-limited condition, which indicates that the electron flow was altered as observed in *M.* *marburgensis*. The *M.* *thermolithotrophicus* genome lacks ElpC, which is a homologue of MvhD and functions as an electron connector in ElpAB–Hdr. In *M.* *thermolithotrophicus*, MvhD is produced constitutively, which suggests that in *Methanococcales* methanogens lacking ElpC, MvhD is used in both Elp–Hdr and Mvh–Hdr complexes. The expression of the nickel-independent pathway for electron flow, combined with the very low levels of expression of MvhAG and FrhAGB under nickel-limited conditions, in two distantly related methanogens, supports the broad conservation and functional relevance of the nickel-independent pathway among CO_2_-reducing hydrogenotrophic methanogens. Our work indicates that methanogens present an extreme example of metal-dependent switching, in which the entire core metabolism is shifted away from [NiFe]-hydrogenases, significantly altering the electron flow in the methanogenic pathway (Fig. 4). Proteomic analysis indicated that the amount of membrane-bound [NiFe]-hydrogenases, Eha and Ehb, is less than 1% of Hmd (Extended Data Fig. 1a). Thus, even when considering anabolic reactions, the contribution of [NiFe]-hydrogenases is small under nickel-limited conditions.

Our experiments indicate that when phylogenetically distant *M.* *marburgensis* and *M.* *thermolithotrophicus* are grown under low nickel concentrations, electrons flow through a pathway that is different from the textbook pathway, in being independent of nickel-based hydrogenases. The conservation of the genes encoding Hmd and Elp in many class I methanogens suggests that methanogens encounter nickel deficiency in natural environments. However, the conservation of the [NiFe]-hydrogenases Frh and Mvh in most methanogens suggests that they also experience nickel-sufficient conditions. Thus, many methanogens seem to be able to adapt to changing bioavailability of nickel by rerouting electron flow between these two pathways. Future transcriptomic studies of environmental samples with known biologically available nickel concentrations will help in understanding the contribution of the non-nickel Hmd system in nature.

## Methods

### Materials

*M.* *marburgensis* DSM 2133 and *M.* *thermolithotrophicus* DSM 2095 were purchased from the German Collection of Microorganisms and Cell Cultures (DSMZ). Most chemicals were from Sigma-Aldrich. H_4_MPT, methenyl-H_4_MPT^+^ and F_420_ were isolated from the *M.* *marburgensis* cells as described previously^34^. Methylene-H_4_MPT was chemically prepared from H_4_MPT with formaldehyde as previously described^35^. F_420_-dependent methylene-H_4_MPT dehydrogenase (Mtd) from *Archaeoglobus fulgidus* was purified from recombinant *Escherichia coli* cells as reported previously^18^. CoM-S-S-CoB was synthesized as described previously^4,19,36^.

### Cultivation methods

The standard medium for culture of *M.* *marburgensis* contains 6.8 g l^−1^ (50 mM) KH_2_PO_4_, 2.544 g l^−1^ (24 mM) Na_2_CO_3_, 2.12 g l^−1^ (40 mM) NH_4_Cl, 0.2 mM MgCl_2_·6H_2_O, 50 µM FeCl_2_·4H_2_O, 5 μM NiCl_2_·6H_2_O, 1 µM CoCl_2_·6H_2_O, 1 µM NaMoO_4_·2H_2_O and 0.09 g l^−1^ Titriplex I. A concentrated trace element solution containing 0.2 M MgCl_2_·6H_2_O, 50 mM FeCl_2_·4H_2_O, 5 mM NiCl_2_, 1 mM CoCl_2_·6H_2_O, 1 mM NaMoO_4_·2H_2_O and 90 g l^−1^ Titriplex I was prepared separately and adjusted to pH 6.7 by addition of NaOH. The trace element solution (0.1% v/v) was added to the medium. To prepare the nickel- or iron-limiting medium, NiCl_2_·6H_2_O and FeCl_2_·4H_2_O were omitted from the trace element solution, and each metal ion concentration in the medium was controlled by addition of 50 mM FeCl_2_·4H_2_O or 5 mM NiCl_2_·6H_2_O solution to the medium. A 0.2% water solution of resazurin sodium salt was finally added to the medium (final concentration 0.6 mg l^−1^).

We used a 360-ml glass fermenter for the cultivation of *M.* *marburgensis* under the controlled nickel concentrations^21^. For cultivation, 80% H_2_/20% CO_2_/0.2% H_2_S mixed gas was supplied by a glass sparger (400 ml min^−1^) without overpressure as described previously. For the H_2_/CO_2_ gas-limiting condition, the mixed gas flow rate was reduced by half. The temperature of the glass vessel was controlled at 65 °C by circulating water from a water bath. The medium was stirred with a plastic stirrer bar at about 300 r.p.m. For the continuous culture, medium was fed by a peristaltic pump with a controlled flow rate. The gas phase of the feed medium was kept under a slight overpressure of N_2_ (less than or equal to about +0.1 bar) to compensate for the outflow of the medium (Supplementary Fig. 2). The cells were collected by anaerobic centrifugation using a Beckman JA-25.50 at 13,000*g* for 15 min at 4 °C.

For cultivation of *M.* *thermolithotrophicus* the standard medium 141 (H_2_/CO_2_) from DSMZ with modifications was used^37^. This medium contains 0.34 g l^−1^ KCl, 4.00 g l^−1^ MgCl_2_·6H_2_O, 3.45 g l^−1^ MgSO_4_·7H_2_O, 0.25 g l^−1^ NH_4_Cl, 0.14 g l^−1^ CaCl_2_·2H_2_O 0.14 g l^−1^, K_2_HPO_4_, 18 g l^−1^ NaCl, 2 ml l^−1^ of 1 g l^−1^ Fe(NH_4_)_2_(SO_4_)_2_·6H_2_O, 1 g l^−1^ sodium acetate, 0.5 ml l^−1^ sodium resazurin (0.1% w/v), 5 g l^−1^ NaHCO_3_, 0.5 g l^−1^ l-cysteine HCl·H_2_O, modified Wolin’s mineral solution without nickel 10 ml l^−1^, and Wolin’s vitamin solution 10 ml. pH was adjusted to 6.8–7.0. Modified Wolin’s mineral solution without nickel contains: 1.5 g l^−1^ nitrilotriacetic acid, 3 g l^−1^ MgSO_4_·7H_2_O, 0.5 g l^−1^ MnSO_4_·H_2_O, 1 g l^−1^ NaCl, 0.1 g l^−1^ FeSO_4_·7H_2_O, 0.18 g l^−1^ CoSO_4_·7H_2_O, 0.1 g l^−1^ CaCl_2_·2H_2_O, 0.18 g l^−1^ ZnSO_4_·7H_2_O, 0.01 g l^−1^ CuSO_4_·5H_2_O, 0.02 g l^−1^ AlK(SO_4_)_2_·12H_2_O, 0.01 g l^−1^ H_3_BO_3_, 0.01 g l^−1^ Na_2_MoO_4_·2H_2_O, 0.3 mg l^−1^ Na_2_SeO_3_·5H_2_O, 0.4 mg l^−1^ Na_2_WO_4_·2H_2_O. Wolin’s vitamin solution contains: 2 mg l^−1^ biotin, 2 mg l^−1^ folic acid, 10 mg ^−1^ pyridoxine hydrochloride, 5 mg l^−1^ thiamine HCl, 5 mg l^−1^ riboflavin, 5 mg l^−1^ nicotinic acid, 5 mg l^−1^ calcium d-(+)-pantothenate, 0.1 mg l^−1^ vitamin B_12_, 5 mg l^−1^ *p*-aminobenzoic acid, 5 mg l^−1^ (dl)-α-lipoic acid. For the nickel-sufficient culture, 1 ml of NiCl_2_·6H_2_O (5 mM) was added to 1 l of medium (final Ni^2+^ concentration = 5 μM). In the nickel-limited culture, 10 ml of NiCl_2_·6H_2_O (5 μM) was added (final Ni^2+^ concentration = 50 nM). *M. thermolithotrophicus* was cultivated in a 100-ml vial sealed with a rubber stopper containing 20 ml liquid medium under a gas phase of 80% H_2_/20% CO_2_ (with +0.5 bar overpressure) at 65 °C with shaking (150 r.p.m.). The gas phase was replaced with a fresh gas mixture every 12 h. Three successive transfers of 5% inoculum to the culture medium containing 5 μM or 50 nM nickel were made from the culture medium containing 5 μM nickel. Cells from the third nickel-sufficient (Ni^2+^ = 5 μM) and nickel-limited (Ni^2+^ = 50 nM) cultures each in triplicate were collected to be used for proteomic analysis.

### Preparation of cell extract

All steps were performed anaerobically in an anaerobic chamber under 3–5% H_2_ in N_2_ (Coy Laboratories). The frozen or fresh *M.* *marburgensis* cells (3.5 g) were suspended in 10.5 ml 50 mM Tris/HCl pH 7.6 containing 2 mM dithiothreitol. The cell suspension was subjected to ultrasonication on ice/water for 2 min using a SONOPULS GM200 (Bandelin) with a 72D tip with 30% cycles 12 times, with 2-min breaks between sonication cycles. The supernatant was collected by centrifugation in a Sorvall WX Ultra centrifuge (Thermo Fisher Scientific) with a T-880 rotor at 41,000 r.p.m. for 30 min at 4 °C. The supernatant (cell extract) contained 150 mg protein (11 mg ml^−1^). For the enzyme assay shown in Fig. 2c, the small molecules were removed from the cell extract by three successive rounds of ultrafiltration (10-kDa cutoff) and dilution. This is referred to as the washed cell extract.

### Enzyme assays

#### Hmd activity

Hmd activity was determined by recording the formation of methenyl-H_4_MPT^+^ at *A*_336nm_ by dehydrogenation of methylene-H_4_MPT under N_2_ (refs. ^35,38^). For the dehydrogenation assay, 0.68 ml of 120 mM potassium phosphate buffer pH 6.0 containing 1 mM EDTA was preincubated in a 1-ml quartz cuvette (1-cm light path) at 40 °C for 5 min. Typically, 10 µl of 1.4 mM methylene-H_4_MPT was added as the substrate to the cuvette to give a 20 µM final concentration. The enzyme reaction was started by addition of 10 µl of (typically 50-fold) diluted cell extract at 40 °C. The activity was calculated using the extinction coefficient of methenyl-H_4_MPT^+^ at 336 nm (21.6 mM^−1^ cm^−1^)^39^. One unit of the enzyme activity is defined as the formation or consumption of 1 µmol of methenyl-H_4_MPT^+^ per minute.

#### Mtd activity

Mtd activity was determined by recording the formation of methenyl-H_4_MPT^+^ at *A*_336nm_ by dehydrogenation of methylene-H_4_MPT in the presence of F_420_ under N_2_ (ref. ^21^), in which the Hmd activity was fully inhibited by addition of an Hmd-specific inhibitor, TosMIC. For the assay, 0.66 ml of 120 mM potassium phosphate buffer pH 6.0 containing 1 mM EDTA was preincubated at 40 °C for 5 min. Typically, 7 µl of 100 µM TosMIC, 10 µl of 1.4 mM methylene-H_4_MPT and 10 µl of 1.4 mM F_420_ were added as substrate to the 1-ml quartz cuvette (1-cm light path) to give 20 µM final concentration each of methylene-H_4_MPT and F_420_. The enzyme reaction was started by addition of 10 µl of (typically 50-fold) diluted cell extract at 40 °C. The activity was calculated using the extinction coefficient of methenyl-H_4_MPT^+^ at 336 nm (21.6 mM^−1^ cm^−1^). One unit of the enzyme activity is defined as dehydrogenation of 1 µmol of methylene-H_4_MPT per minute.

#### Frh activity

Frh activity was determined by recording the reduction of F_420_ at *A*_401nm_ under H_2_ (+0.4 bar)^21^. For the assay, 0.67 ml of 50 mM Tris/HCl pH 7.6 containing 10 mM dithiothreitol was preincubated at 55 °C for 5 min. Typically, 9 µl of 1.4 mM F_420_ was added as substrate to a 1-ml quartz cuvette (1-cm light path) to give 18 µM final concentration, and then 10 µl of 3.5 mM sodium dithionite was added to give a 50 µM final concentration. The enzyme activity was started by addition of 10 µl of (typically 20-fold) diluted cell extract at 55 °C. For dilution of the cell extract for the Frh assay, we used 50 mM Tris/HCl pH 7.6 containing 25 µM FAD. The activity was calculated using the extinction coefficient of F_420_ at 401 nm (25.9 mM^−1^ cm^−1^)^39^. One unit of enzyme activity is defined as reduction of 1 µmol of F_420_ per minute.

#### Mvh activity

Mvh activity was determined by recording the reduction of methyl viologen (MV) under H_2_ (+0.4 bar)^21^. For the assay, 0.67 ml of 50 mM Tris/HCl pH 7.6 containing 2 mM dithiothreitol was preincubated at 65 °C for 5 min. Typically, 7 µl of 200 mM MV was added as the substrate to a 1-ml quartz cuvette (1-cm light path) to give a 2 mM final concentration, and then 10 µl of 20 mM sodium dithionite was added to give a 290 µM final concentration to ensure the anaerobic condition. By addition of 10 µl of (typically 100-fold) diluted cell extract, the enzyme reaction was started at 65 °C. The activity was calculated using the extinction coefficient of MV at 604 nm (13.7 mM^−1^ cm^−1^)^21^. One unit of enzyme activity is defined as reduction of 2 µmol of MV per minute.

#### Benzyl-viologen-dependent Hdr activity

Benzyl-viologen-dependent Hdr (BV Hdr) activity was determined by recording the oxidation of reduced BV by heterodisulfide (CoM-S-S-CoB)^3^. For the assay, 0.7 ml of 800 mM potassium phosphate buffer pH 7.0 was preincubated at 65 °C for 5 min. A 7 µl volume of 200 mM BV was added to a 1-ml quartz cuvette (1-cm light path) to give a 2 mM final concentration, and then 10 µl of 20 mM sodium dithionite was added. A 10 µl volume of cell extract was added to the vial. The enzyme reaction was started by addition of 7 µl of 100 mM CoM-S-S-CoB to give a 1 mM final concentration at 65 °C. The activity was calculated using the extinction coefficient of BV at 578 nm (8.6 mM^−1^ cm^−1^)^21^. One unit of enzyme activity is defined as oxidation of 2 µmol of BV per minute.

#### H_2_-dependent Hdr activity

H_2_:CoM-S-S-CoB oxidoreductase activity was determined by monitoring the formation of thiols (CoM-SH and CoB-SH) from CoM-S-S-CoB using H_2_ as reductant^3^. For the assay, 1.0 ml of 1.6 M potassium phosphate buffer pH 7.0 was preincubated in a 5-ml amber vial at 65 °C for 5 min under H_2_ (+0.2 bar). A 50 µl volume of cell extract was added to the vial. The enzyme reaction was started by addition of 7 µl of 100 mM CoM-S-S-CoB (1 mM final concentration). A 100 µl aliquot of the reacted sample was diluted with 900 µl of 100 mM sodium phosphate buffer pH 8.0, to which 18 µl of 4 mg ml^−1^ 5,5′-dithiobis(2-nitrobenzoic acid) (Ellman reagent) was added and incubated at 25 °C for 10 min. The formation of thiol was calculated from the extinction coefficient of the Ellman reagent at 412 nm (14 mM^−1^ cm^−1^)^18^. One unit of enzyme activity is defined as the formation of 2 µmol of thiol per minute.

#### F_420_H_2_-dependent Hdr activity

F_420_H_2_:CoM-S-S-CoB oxidoreductase (F_420_ Hdr) activity was determined by monitoring the formation of thiols (CoM-SH and CoB-SH) from CoM-S-S-CoB using F_420_H_2_ formed by the Hmd+Mtd coupled reaction with H_2_ as the reductant in the assay. For the assay, 1.0 ml of 800 mM potassium phosphate buffer pH 7.0 containing 20 μM methenyl-H_4_MPT^+^ and 20 µM F_420_ was preincubated in a 1-ml quartz cuvette (1-cm light path) at 65 °C for 5 min under H_2_. After addition of 10 µl of 5 U ml^−1^ Hmd from *M.* *marburgensis*^35^ and 10 μl of 5 U ml^−1^ Mtd from *A.* *fulgidus*^18^, the assay solution was incubated at 65 °C for 5 min. Conversion of F_420_ to F_420_H_2_ was confirmed by monitoring absorbance at 401 nm, and then 7 µl of 100 mM CoM-S-S-CoB solution was added to the solution (1 mM final concentration). The enzyme reaction was started by addition of 50 µl of enzyme solution. A 100-µl aliquot of the reacted sample was diluted with 900 µl of 100 mM sodium phosphate buffer pH 8.0, to which 18 µl of 4 mg ml^−1^ Ellman reagent was added and incubated at 25 °C for 10 min. The formation of thiols was calculated from the extinction coefficient of the Ellman reagent at 412 nm (14 mM^−1^ cm^−1^). One unit of enzyme activity is defined as the formation of 2 µmol thiol per minute.

#### Fdh activity

Fdh activity was determined by monitoring the reduction of BV in the presence of sodium formate^18^. For the assay, 0.6 ml of 50 mM potassium phosphate buffer pH 7.0 was preincubated in a 1-ml quartz cuvette (1-cm light path) at 40 °C for 5 min. A 7 µl volume of 200 mM BV was added as the substrate to the cuvette to give a 2 mM final concentration. A 5 µl volume of 10 mM sodium dithionite was added followed by 10 µl 10-fold diluted cell extract. The reaction was started by addition of 70 µl of 20 mM sodium formate. The activity was calculated using the extinction coefficient of BV at 578 nm (8.6 mM^−1^ cm^−1^). One unit of enzyme activity is defined as the reduction of 2 µmol of BV per minute. As a positive control, the cell extract of *M.* *maripaludis* Mm1328 was used^40^, which contains FdhAB-type formate dehydrogenase^17^.

### Additional notes on the enzyme activity data shown in Fig. 2

The data in Fig. 2a are consistent with published data and the proteomic data in Fig. 1. The data in Fig. 2b,c are in agreement with the growth rate and the proteomic data shown in Fig. 1. On the basis of these findings, we predicted the presence of the Elp–Hdr–Fmd complex, and this hypothesis was supported by the Hmd+Mtd-dependent Hdr reaction with F_420_H_2_ as shown in Fig. 2e. This was also supported by the purification and the cryo-EM analysis of the enzyme complex.

### Proteomic analysis

In the proteomic analysis of *M.* *marburgensis*, the three cell samples were obtained at three different times from the stable continuous culture. In the case of proteomic analysis of *M.* *thermolithotrophicus*, the proteomic samples were obtained from three independent batch cultures under controlled nickel concentrations.

The cell pellets were lysed with 2% sodium N-lauroylsarcosinate at 90 °C and additionally sonicated. The protein concentration was subsequently measured using the bicinchoninic acid method. Carbamidomethylation of the cysteines was performed using 5 mM tris(2-carboxyethyl)phosphine/100 mM ammonium bicarbonate at 90 °C for 10 min and 10 mM iodoacetamide at 25 °C for 30 min. Then 50-µg aliquots of the samples were diluted to 0.5% sodium N-lauroylsarcosinate and digested overnight at 30 °C with trypsin, mass spectrometry (MS)-approved (Serva). Before liquid chromatography–MS analysis, samples were desalted using a Chromabond Spin C18 WP cartridge (Macherey-Nagel) according to the manufacturer’s instructions. Dried and reconstituted peptides were then analysed using liquid chromatography–MS carried out on an Orbitrap Exploris 480 instrument connected to an Ultimate 3000 RSLC nano and a nanospray ion source (Thermo Scientific). Peptide separation was performed on a reverse-phase high-performance liquid chromatography column (75 μm × 42 cm) packed with C18 resin (2.4 μm; Dr. Maisch) run with a 60-min gradient (0.15% formic acid/2% acetonitrile to 0.15% formic acid/50% acetonitrile). MS data were searched against an in-house *M.* *marburgensis* protein database using SEQUEST HT embedded into Proteome Discoverer 1.4 software (Thermo Scientific). In the case of the analysis of the protein fraction of the size-exclusion chromatography, the purified fraction was directly used for the MS-based analysis. Proteomic data were quantified using DIA-NN 1.8 software^41^. To compare the intensity between different proteins, we calculated intensity-based absolute quantification values^42^.

### Protein purification

The frozen *M.* *marburgensis* cells (3.5 g) were suspended in 10.5 ml 50 mM Tris/HCl pH 7.6 containing 2 mM dithiothreitol and disrupted as described above. After centrifugation in a Sorvall WX Ultra centrifuge with a T-880 rotor at 41,000 r.p.m. for 30 min at 4 °C, the supernatant containing 150 mg protein, was loaded on a HiTrap Q-HP (5 ml) column, which was equilibrated with 50 mM Tris/HCl pH 7.6 containing 2 mM dithiothreitol (buffer A). The proteins bound on the column were eluted with a step gradient of 50 mM Tris/HCl pH 7.6 containing 2 mM dithiothreitol and 1 M NaCl (buffer B). The step gradient was 30%, 40%, 44%, 48%, 52%, 56%, 60% and 100% buffer B at a 2 ml min^−1^ flow rate. The 48% or 52% buffer B fraction of the Q-Sepharose chromatography containing most of the Hdr activity was collected and diluted with the same volume of 50 mM Tris/HCl pH 7.6 containing 2 mM dithiothreitol and 1.2 M ammonium sulfate (buffer C). The diluted sample was loaded on a HiTrap Phe-HP (5 ml) column equilibrated with buffer C. The proteins bound on the column were eluted with a step gradient of 50%, 58%, 67%, 83% and 100% buffer A at a 2 ml min^−1^ flow rate. The elution conditions of Q-Sepharose and Phe-Sepharose columns are according to the previous method used for purification of the Mvh–Hdr complex from *M.* *marburgensis*^26^. The 67% buffer A fraction was exchanged into buffer containing 2 mM dithiothreitol and 150 mM NaCl by an Amicon Ultra-0.5 (3-kDa cutoff) filter. The sample was finally concentrated to about 0.5 ml and applied to a Superose 6 Increase (10/300 GL) size-exclusion column and eluted at a flow rate of 0.5 ml min^−1^. The eluate was collected in 0.5-ml fractions. The size-exclusion column was calibrated with thyroglobulin (bovine) 670 kDa, γ-globulin (bovine) 158 kDa, ovalbumin (chicken) 44 kDa, myoglobin (horse) 17 kDa, and vitamin B_12_ 1.35 kDa. The standard materials were from Bio-Rad.

Typically, the cell extract containing 150 mg protein with 150 U of BV Hdr activity was fractionated on a HiTrap Q-HP column as described above. The proteins with BV Hdr activity eluted mainly in the 0.52-M NaCl step gradient from the HiTrap Q-HP column. The total yield of BV Hdr activities in the HiTrap Q-HP fractions was 98 U (65% of the loaded sample). The main Hdr fraction at 0.52-M NaCl containing 5.2 mg of protein with 43 U of BV Hdr activity was further fractionated with a HiTrap Phe-HP column, in which the BV Hdr activity was eluted in step gradients containing 0.5-M, 0.4-M and 0.2-M ammonium sulfate as reported previously for the purification of the Mvh–Hdr–Fmd complex from *M.* *marburgensis*^19^. The total yield of BV Hdr activity in the three phenyl-Sepharose fractions was 36 U (84% of the loaded sample). We used the 0.4-M ammonium sulfate fraction containing 0.9 mg protein with 15 U BV-Hdr activity for further fractionation on a Superose 6 Increase column. The elution profile of the BV Hdr and F_420_ Hdr activity and the profile of the F_420_-dependent Hdr activity are shown in Fig. 2f, and SDS–polyacrylamide gel electrophoresis (PAGE) analysis is shown in Extended Data Fig. 2 and Supplementary Fig. 7. The fractions with BV Hdr activity from the Superose 6 Increase contained 0.68 mg protein and 11 U BV Hdr activity (73% of the loaded sample). The Elp–Hdr–Fmd complexes were purified seven times. We performed SDS–PAGE analysis four times and proteomic analysis once (Extended Data Table 1 and Extended Data Fig. 3a–d). The SDS–PAGE data supported the reproducibility of the purification and the proteomic analysis.

### Cryo-EM sample preparation and data collection

The cryo-EM sample preparation was performed immediately after the Superose 6 Increase (10/300 GL) purification step inside an anaerobic chamber (<1 ppm O_2_; Coy Laboratory Products). The fraction corresponding to the 1-MDa peak was used for freezing. For each grid (glow-discharged UltrAuFoil 1.2/1.3 300 mesh), 3 µl of the sample (1 mg ml^−1^) was applied, blotted for 4 s with Whatman 595 filter paper (Sigma-Aldrich) at 4 °C under 100% humidity, and plunge-frozen in liquid ethane using a Vitrobot Mark IV (Thermo Fisher Scientific). The dataset was corrected using aberration-free image shift at a Titan Krios G3i equipped with a BioQuantum energy filter and a K3 detector (Gatan) at an image pixel size of 0.837 Å per pixel. Dose-fractionated videos were collected, with a total dose of 65 electrons per square ångström spread over 65 fractions and a defocus range between −0.8 µm and −2.4 µm. EPU v3.6 (Thermo Fisher Scientific) was used for automated data acquisition of 7,768 videos.

### Image processing and model building

A detailed workflow of the steps for data processing is shown in Extended Data Fig. 4. An initial fast data screening was performed on-the-fly using CryoSPARC Live^43^. Videos were motion-corrected and defocus parameters were calculated using the Patch Motion correction and CTF estimation tools, respectively. Corrected micrographs were then selected on the basis of a maximum 4.0 Å CTF resolution estimate, and micrographs with substantial crystalline ice contamination were manually removed. A total of 6,488 curated micrographs were then exported to CryoSPARC v3.1 (ref. ^43^) for data processing. Blob picker, template picker and a trained Topaz model^44^ were used one after another to reach optimal particle picking. An initial set of particles was obtained using the Blob picker (minimum and maximum diameters of 60 Å and 550 Å, 500 local maxima considered), and the extracted particles (2.7 million particles, 500-pixel box downsampled to 126 pixels) were subjected to two rounds of 2D classification. A total of 31 class averages from 351,000 particles were selected and used as templates for the template picker. The initially picked particles (551,000) were extracted (500-pixel box, downsampled to 150 pixels) and subjected to two rounds of 2D classification to remove bad particles. A total of 350,000 particles corresponding to 110 2D classes were then randomized, and a subset of 20,000 was used to train a Topaz model (downsampling factor 8, 500 expected particles per micrograph, ResNet8). The Topaz Extract tool (radius 15, 200 iterations, downsampling 8) was used to pick 1.3 million particles that were extracted (416-pixel box, no downsampling) and used for 2D classification. A total of 1.1 million particles corresponding to 108 classes were selected for further processing. Three ab initio models were obtained using the ab initio reconstruction job and used as templates for heterogeneous refinement (*C*_1_, no downsampling). The map of one of the three classes showed a clear density for Hdr(ABC)_2_ and blurred regions for the flexible Elp arms. No density could be observed for Fmd.

To continue with the processing using RELION 4 (ref. ^45^), particle coordinates of 493,925 particles belonging to the good class from heterogeneous refinement were exported to RELION format using pyem^46^. Before particle extraction, the raw videos were motion-corrected and dose-weighted with RELION’s MotionCor2 implementation^47^ using 5 × 5 patches, and CTF resolution was estimated using CTFFind4.1 (ref. ^48^). The particles were extracted in a box of 416 pixels, downsampled to 384 pixels and reimported into CryoSPARC. A masked 3D refinement was performed with *C*_2_ symmetry, giving a map at 2.48 Å resolution. CTF parameters were refined using local and global CTF refinement tools, and a map of Hdr(ABC)_2_ (without the N- and C-terminal flexible HdrA domains forming part of the flexible Elp arms) could be obtained at 2.04 Å after homogeneous refinement with *C*_2_ symmetry applied. Data processing was then performed separately for the Hdr(ABC)_2_ region and the flexible Elp arms.

For the Elp arms, the *C*_2_-refined particles (493,925) were converted to the Relion format using Pyem and then symmetry-expanded using relion_particle_symmetry_expand. A model of the Fdh–Hdr–Fmd complex from *M.* *hungatei* (PDB accession code 7BKC)^18^ was aligned to the Hdr(ABC)_2_ map, Fmd subunits were deleted, and a 30-Å low-pass-filtered volume was generated using the molmap tool of ChimeraX^49^. The region corresponding to the FdhAB–MvhD mobile arm was used to create a 30 Å low-pass-filtered mask. The reference volume and mask were used for a focused 3D classification of the symmetry-expanded particles without alignment (*T* = 4, 3 classes, 25 iterations). The three classes obtained corresponded to the Hdr region without any apparent density for the mobile arm (676,764 particles), and two clearly different states of the Elp arm (state 1, 76,528 particles; state 2, 234,858 particles). These conformational states are very similar to conformational states 1 and 2 of the *M.* *hungatei* Fdh–Hdr–Fmd complex^18^. Particles corresponding to each state of the Elp arms were reimported into CryoSPARC for masked local and CTF refinements using masks obtained from the 3D classification output volumes. Then, the particles were reimported into Relion for Bayesian polishing and re-extracted with a 448-pixel box size without downsampling. The particles were reimported into CryoSPARC for final local and CTF refinements. For each conformation, three different maps were obtained: one consensus map obtained using a mask containing the arm and the Hdr region, and two focused maps obtained using masks for the arm (mobile-arm-focused maps), and for the Hdr(ABC)_2_ dimer separately (Hdr-focused maps). For state 1, a map of Elp at 2.4 Å, a map of Hdr at 2.1 Å and a consensus map at 2.47 Å were obtained. For state 2, a map of the arm at 2.2 Å, a map of Hdr at 2.3 Å and a consensus map at 2.3 Å were obtained (Extended Data Fig. 4 and Supplementary Figs. 3 and 4).

For the analysis of MvhB, we used Relion 5.0 (ref. ^50^) to perform focused 3D classification (Blush regularization, *T* = 4, 3 classes, 25 iterations)^51^, of the symmetry-expanded particles without alignment. We used the same reference map as for the mobile arms (see above), and we created a mask (30 Å low-pass filter, 4-pixel extension and 12-pixel soft-padding) from the entire MvhB subunit of the AF3 HdrA–MvhB–FmdF complex (Extended Data Fig. 7) after alignment with the reference map. One of the three classes, corresponding to around 12% of the particles (119,398), showed an extra density that could not be fitted to the inserted ferredoxin-like domain of HdrA. The particles were further refined using 3D refinement with 1.8 Å local and angular searches and Blush regularization. A 3 Å-resolution map could be obtained. The particles were then imported into CryoSPARC v4.5.1 and further subjected to local refinement using a wider mask including HdrA. Then, the particles were subjected to local and global CTF refinements, and a final local refinement was performed (Extended Data Fig. 7c), which resulted in a 2.54 Å map showing an additional density attached to the inserted ferredoxin-like domain of HdrA.

For Hdr(ABC)_2,_ the *C*_2_-refined particles (493,925) were reimported into Relion for Bayesian polishing (448-pixel box size, no downsampling). The particles were reimported into CryoSPARC and used for several rounds of focused local and CTF refinements until a focused map at 1.85 Å resolution was obtained.

The focused maps corresponding to the Hdr(ABC)_2_ map at 1.85 Å and the Elp arm in conformational state 2 at 2.2 Å were used for automatic model building using the machine-learning-based tool ModelAngelo^52^. The program COOT^53^ was then used to place cofactors and to inspect and manually adjust the models. Then, for each conformational state, the models of each region were rigid-body-fitted into the consensus map to generate combined models of Hdr(ABC)_2_ plus one Elp arm (in state 1 or under state 2) in ChimeraX. Finally, composite maps of the subcomplex comprising Hdr(ABC)_2_ plus one Elp arm were generated with the tool phenix.combine_focused_maps^54^ using as inputs: the consensus maps, the focused maps and the combined models. Furthermore, a combined map of the Elp–Hdr dimer was generated using the combined map of Elp–Hdr in state 2 and a dimer model (ElpABC–HdrABC)_2_ in conformational state 2, using the tool phenix.combine_focused_maps. Iterative rounds of PHENIX real-space refinement^55^ and manual inspection and readjustment in COOT were performed to optimize the model stereochemistry and the fit to the cryo-EM density map as assessed with PHENIX, MolProbity^56^ and Q-score^57^. Root mean square deviation values were calculated by the mmaker command of ChimeraX^49^.

### AF structural modelling

The HdrA–MvhB–FmdF complex of *M.* *marburgensis* (Extended Data Fig. 7) was predicted with the AlphaFold3.0 server^33^. The sequences used for the prediction were obtained from the UniProtKB database: *M.* *marburgensis* HdrA (Q50756), MvhB (P60232) and FmdF (D9PU52). Output models were assessed to determine whether a credible complex was generated, and subunit interfaces were inspected manually for surface complementarity and the absence of clashing atoms. When needed, cofactors were added to the predicted models. For HdrA and FmdF subunits, the AF3 models were aligned to experimental models of HdrA (this paper, PDB accession code 8RWN) and FmdF (*Methanothermobacter wolfeii*, PDB accession code 5T61), and cofactors were added to the corresponding positions. For MvhB, [4Fe–4S] clusters were fitted at the predicted [4Fe–4S]-binding sites, which could be identified by the location of the coordinating cysteines.

### Reporting summary

Further information on research design is available in the Nature Portfolio Reporting Summary linked to this article.

## Online content

Any methods, additional references, Nature Portfolio reporting summaries, source data, extended data, supplementary information, acknowledgements, peer review information; details of author contributions and competing interests; and statements of data and code availability are available at 10.1038/s41586-025-09229-y.

## Supplementary information

Supplementary Information Supplementary Figs. 1–7 and Table 1. Supplementary Fig. 1 The reactions catalysed by Frh and the coupled reaction of Hmd and Mtd. The reactions catalysed by F_420_-reducing [NiFe]-hydrogenase (Frh) and the coupled reaction of [Fe]-hydrogenase (Hmd) and F_420_-dependent methylene-H_4_MPT dehydrogenase (Mtd) are shown. Supplementary Fig. 2 Schematic presentation of the continuous culture using continuous flow of the medium. A gas mixture (80% H_2_/20% CO_2_/0.2% H_2_S) was supplied by a glass sparger (400 ml min^−1^). The temperature of the glass vessel was controlled at 65 °C. The medium was stirred at about 300 r.p.m. The medium was fed with a controlled flow rate. Supplementary Fig. 3 CryoEM maps, half-map and map–model Fourier shell correlation (FSC) curves, and local resolution estimates of the state-1 ElpABC–(HdrABC)_2_ complex. a,b, State 1 ElpABC mobile-arm-focused map at 2.4 Å (a), and of Hdr(ABC)_2_ at 2.36 Å (b). c, The consensus map at 2.45 Å obtained by using a mask including the ElpABC mobile arm and the Hdr regions. d,e, Local resolution maps obtained from the ElpABC mobile-arm-focused map (d) and the Hdr(ABC)_2_-focused map (e), shown for clarity over the state-1 Elp–Hdr composite map (grey; Extended Data Figs. 4 and 5a). f, Map versus model FSC curves of the masked and unmasked state-1 Elp–Hdr model (0.5 threshold, black line). g, Orientation distribution maps of each focused 3D-refinement. Supplementary Fig. 4 CryoEM maps, half-map and map–model FSC curves, and local resolution estimates of the state-2 ElpABC-(HdrABC)_2_ complex. a,b, State-2 ElpABC mobile-arm-focused map at 2.2 Å (a), and of Hdr(ABC)_2_ at 2.1 Å (b). c, The consensus map at 2.3 Å obtained using a mask including the ElpABC mobile arm and the Hdr regions. d,e, Local resolution maps obtained from the ElpABC mobile-arm-focused map (d) and the Hdr(ABC)_2_-focused map (e), shown for clarity over the state-2 composite map (grey; Extended Data Figs. 4 and 5a). f, Map versus model FSC curves of the masked and unmasked state-2 Elp–Hdr model (0.5 threshold, black line). g, Orientation distribution maps of each focused 3D refinement. Supplementary Fig. 5 Densities and models of the non-cubane iron–sulfur clusters of ElpB HB1 and HB2, the cubane iron–sulfur cluster of HdrC HC2, the cubane iron–sulfur cluster of HdrA HA3, three cubane iron–sulfur clusters of HdrA′ (HA4′, HA5′ and HA6′), the bifurcating FAD of HdrA′ (FAD′), the His325-coordinated cubane iron–sulfur cluster of ElpB (EB3), the FAD of ElpB and the ElpC [2Fe–2S] cluster (EC). Supplementary Fig. 6 The site of CoB-S-S-CoM reduction in HdrB (a–c) and the FAD-binding site (d–f) exhibit a very similar conformation in Elp–Hdr (a), Fdh–Hdr–Fmd in *M. hungatei* (PDB accession code 7BKC) (b) and Mvh–Hdr of *M. thermolithotrophicus* (PDB accession code 5ODH) (c). The cubane iron–sulfur clusters of HdrC (HC1 and HC2), the non-cubane iron–sulfur clusters of HdrB (HB1 and HB2) and the HdrA-bound FAD (FAD) and the amino acids involved in FAD binding are shown as sticks. Supplementary Fig. 7 Raw and uncropped data for gels used for Extended Data Fig. 2. Supplementary Table. 1 Presence or absence of the genes encoding Hmd, ElpAB, FdhAB and MvhD homologues. Only representative methanogens are listed. The presence or absence of the genes encoding the respective proteins is indicated by a plus (or the protein annotation number with amino acid length, AA) or minus sign.

Reporting Summary

Supplementary Data 1 Source data for all values.

Supplementary Data 2 Source data for proteomic data of the fractions in Fig. 2f.

## Source data

Source Data Fig. 1 and Source Data Extended Data Fig. 1

Source Data Fig. 2

Source Data Extended Data Fig. 3

Source Data Extended Data Fig. 8

## Figures and Tables

**Extended Data Table 1:**
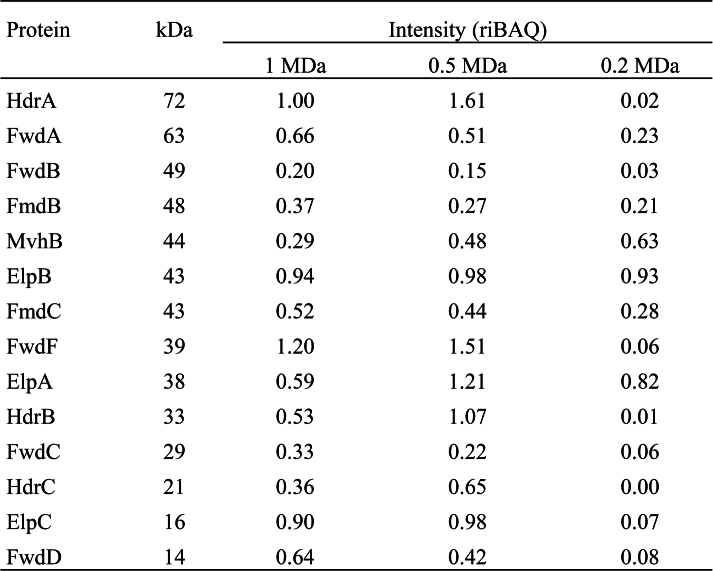
Proteomic data of the 1-MDa, 0.5-MDa and 0.2-MDa fractions shown in Fig. 2f

The intensity of the protein signals is indicated by the relative ratio of iBAQ to that of HdrA in the 1-MDa fraction (riBAQ). Single proteomic analysis of each fraction was performed. The reliability of these values is supported by the protein profiles observed by SDS-PAGE, which was performed four times.

**Extended Data Table 2:**
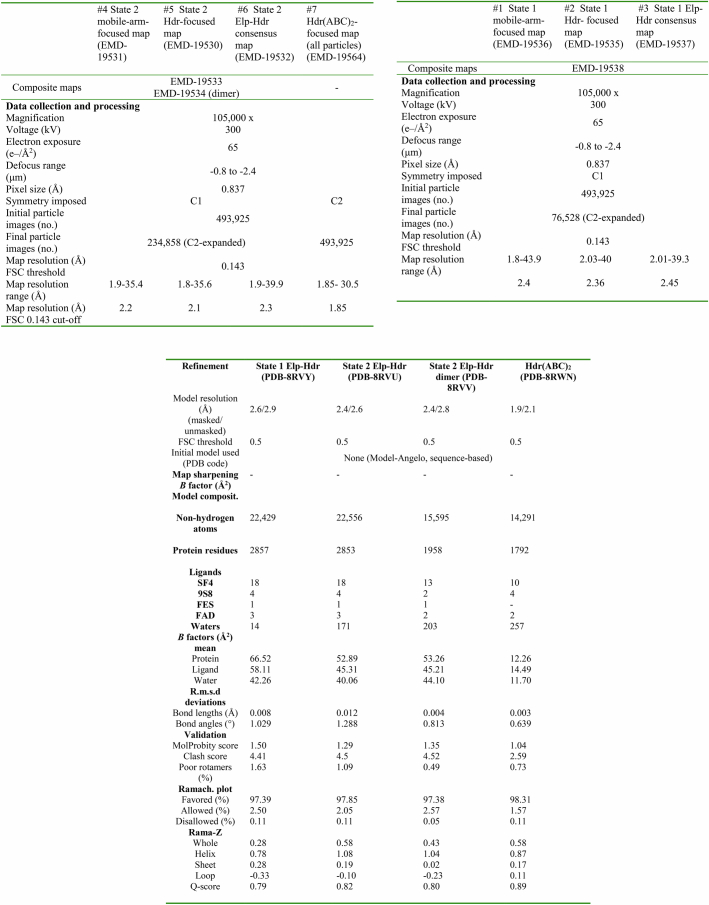
Cryo-EM data collection, refinement and validation statistics

## Data Availability

The MS proteomics data have been deposited to the ProteomeXchange Consortium via the PRIDE^58^ partner repository with the dataset identifiers PXD063927 (Extended Data Table 1 and Extended Data Fig. 3a–d) and PXD063936 (Fig. 1 and Extended Data Figs. 1 and 8b). The state-1 Elp–Hdr model has been deposited in the PDB with the accession code 8RVY, and the composite map has been deposited in the Electron Microscopy Data Bank (EMDB) with the accession code EMD-19538. This composite map is derived from the associated focused maps with the EMDB accession codes EMD-19536 (state-1 mobile-arm-focused map) and EMD-19535 (state-1 Hdr-focused map) and the consensus map with the EMDB accession code EMD-19537 (state 1 Elp–Hdr consensus map). The state-2 Elp–Hdr asymmetric model has been deposited in the PDB with the accession code 8RVU, and the composite map has been deposited in the EMDB with the accession code EMD-19533; the state-2 dimer model has been deposited in the PDB with the accession code 8RVV, and the dimer composite map has been deposited in the EMDB with the accession code EMD-19534. These composite maps are derived from the associated focused maps with the EMDB accession codes EMD-19531 (state-2 mobile-arm-focused map) and EMD-19530 (state-2 Hdr-focused map) and the consensus map with the accession code EMD-19532 (state 2 Elp–Hdr consensus map). Finally, the Hdr(ABC)_2_ model has been deposited in the PDB with the accession code 8RWN, and the map has been deposited in the EMDB with the accession code EMD-19564. SEQUEST, Proteome Discoverer 1.4 and DIA-NN were used for proteomic analysis. The sequences used for the prediction were obtained from the UniProtKB database: *M.* *marburgensis* HdrA (Q50756), MvhB (P60232) and FmdF (D9PU52). Source data are provided with this paper.
