# Supplementary material for: Electron flow in hydrogenotrophic methanogens under nickel limitation
Source: Nature. 2025 Jul 2;644(8076):490–6. doi: 10.1038/s41586-025-09229-y (PMC12350162; doi:10.1038/s41586-025-09229-y)
Supplement: Supplementary file 1 — Supplementary Figs. 1–7 and Table 1. Supplementary Fig. 1 The reactions catalysed by Frh and the coupled reaction of Hmd and Mtd. The reactions catalysed by F420-reducing [NiFe]-hydrogenase (Frh) and the coupled reaction of [Fe]-hydrogenase (Hmd) and F420-dependent methylene-H4MPT dehydrogenase (Mtd) are shown. Supplementary Fig. 2 Schematic presentation of the continuous culture using continuous flow of the medium. A gas mixture (80% H2/20% CO2/0.2% H2S) was supplied by a glass sparger (400 ml min−1). The temperature of the glass vessel was controlled at 65 °C. The medium was stirred at about 300 r.p.m. The medium was fed with a controlled flow rate. Supplementary Fig. 3 CryoEM maps, half-map and map–model Fourier shell correlation (FSC) curves, and local resolution estimates of the state-1 ElpABC–(HdrABC)2 complex. a,b, State 1 ElpABC mobile-arm-focused map at 2.4 Å (a), and of Hdr(ABC)2 at 2.36 Å (b). c, The consensus map at 2.45 Å obtained by using a mask including the ElpABC mobile arm and the Hdr regions. d,e, Local resolution maps obtained from the ElpABC mobile-arm-focused map (d) and the Hdr(ABC)2-focused map (e), shown for clarity over the state-1 Elp–Hdr composite map (grey; Extended Data Figs. 4 and 5a). f, Map versus model FSC curves of the masked and unmasked state-1 Elp–Hdr model (0.5 threshold, black line). g, Orientation distribution maps of each focused 3D-refinement. Supplementary Fig. 4 CryoEM maps, half-map and map–model FSC curves, and local resolution estimates of the state-2 ElpABC-(HdrABC)2 complex. a,b, State-2 ElpABC mobile-arm-focused map at 2.2 Å (a), and of Hdr(ABC)2 at 2.1 Å (b). c, The consensus map at 2.3 Å obtained using a mask including the ElpABC mobile arm and the Hdr regions. d,e, Local resolution maps obtained from the ElpABC mobile-arm-focused map (d) and the Hdr(ABC)2-focused map (e), shown for clarity over the state-2 composite map (grey; Extended Data Figs. 4 and 5a). f, Map versus model FSC curves of the masked and unmaske [file 41586_2025_9229_MOESM1_ESM.pdf]

nature portfolio

https://doi.org/10.1038/s41586-025-09229-y

Supplementary information

Electron flow in hydrogenotrophic methanogens under nickel limitation

In the format provided by the authors and unedited

Supplementary Information

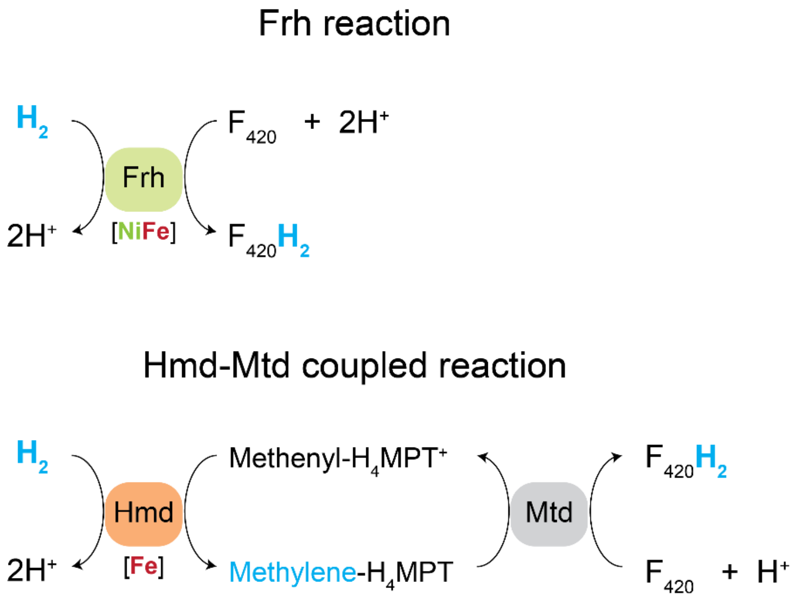

Supplementary Fig. 1 | The reactions catalyzed by Frh and the coupled reaction of Hmd and Mtd. The reactions catalyzed by F_{420}-reducing [NiFe]-hydrogenase (Frh) and the coupled reaction of [Fe]-hydrogenase (Hmd) and F_{420}-dependent methylene-H_4MPT dehydrogenase (Mtd) are shown.

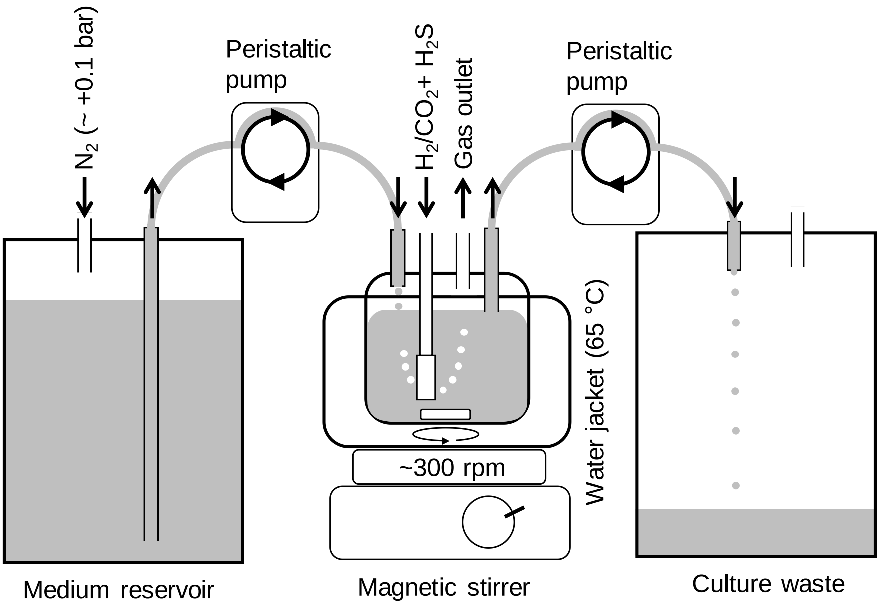

Supplementary Fig. 2 | Schematic presentation of the continuous culture using continuous flow of the medium. A gas mixture (80% H_2 / 20% CO_2 / 0.2% H_2S) was supplied by a glass sparger (400 mL/min). The temperature of the glass vessel was controlled at 65 °C. The medium was stirred at ~300 rpm. The medium was fed with a controlled flow rate.

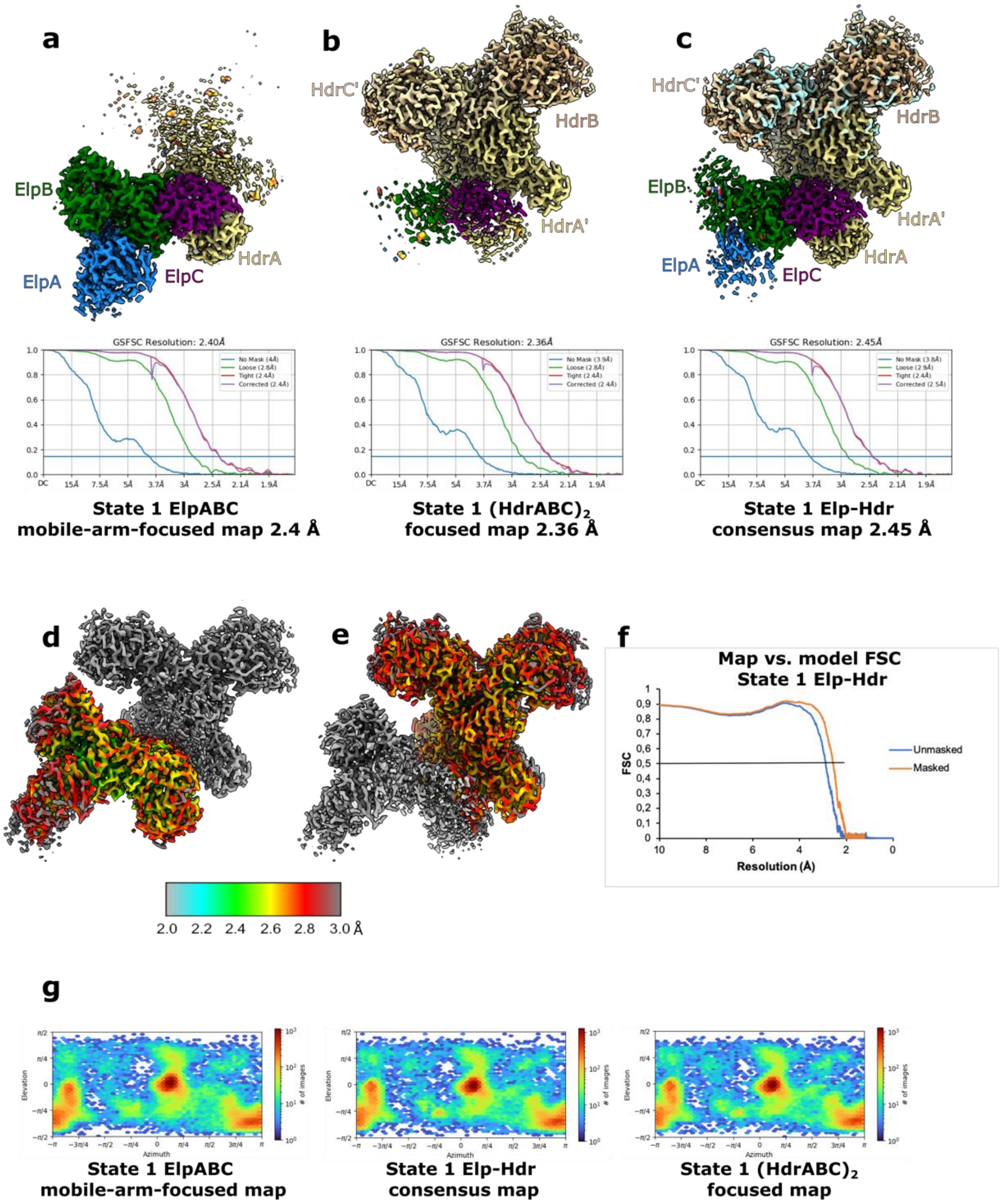

Supplementary Fig. 3 | CryoEM maps, half-map and map-model FSC curves, and local resolution estimates of the State 1 ElpABC-(HdrABC)$_2$ complex. **a & b**, State 1 ElpABC mobile-arm-focused map at 2.4 Å (a), and of Hdr(ABC)$_2$ at 2.36 Å (b). **c**, The consensus map at 2.45 Å obtained by using a mask including the ElpABC mobile arm and the Hdr regions. **d & e**, Local resolution maps obtained from the ElpABC mobile-arm-focused map (d) and the Hdr(ABC)$_2$ focused map (e), displayed for clarity over the State 1 Elp-Hdr composite map (grey, see Extended Data Fig. 4 and 5a). **f**, Map vs. model FSC curves of the masked and unmasked State 1 Elp-Hdr model (0.5 threshold, black line). **g**, Orientation distribution maps of each focused 3D-refinement.

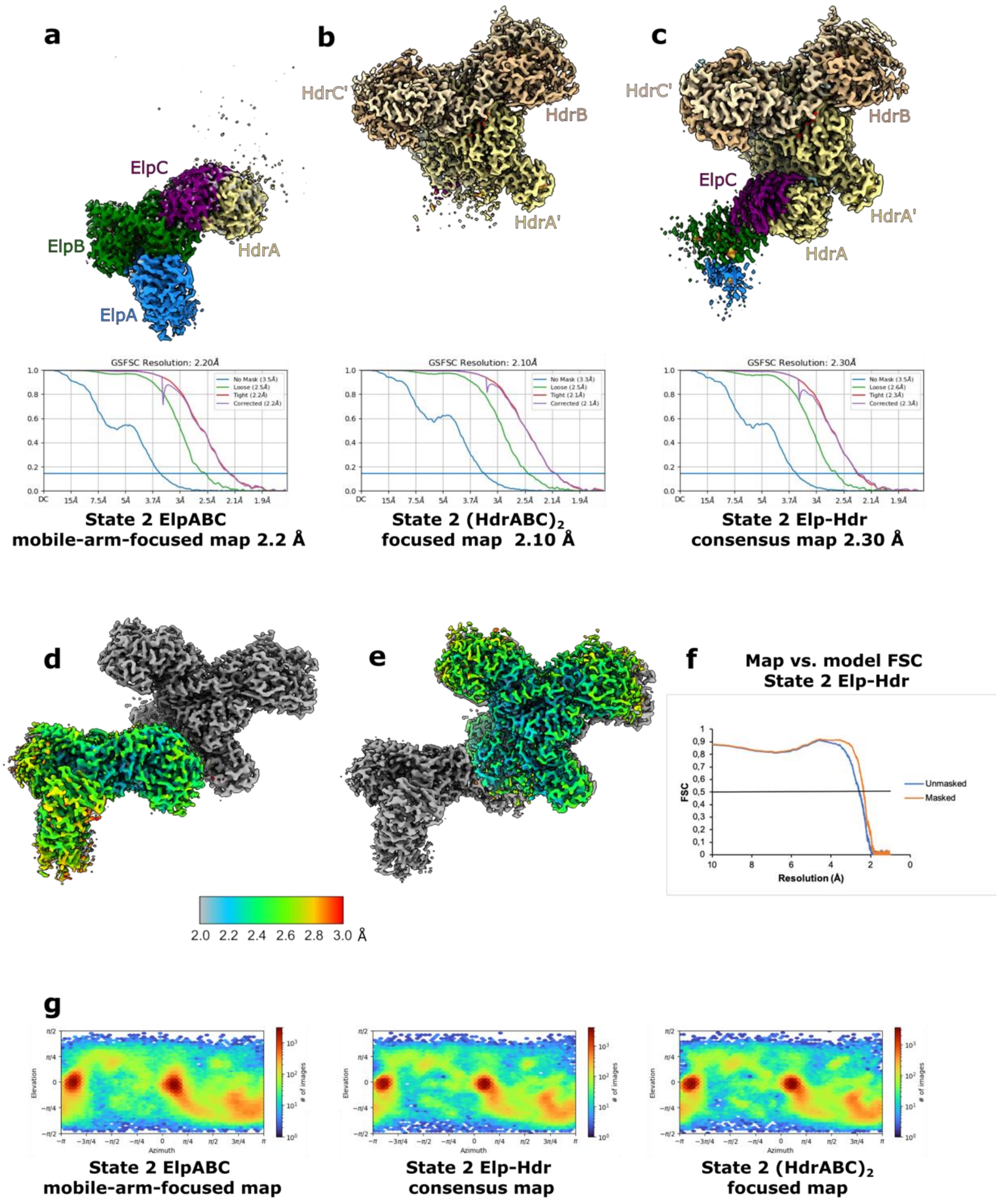

Supplementary Fig. 4 | CryoEM maps, half-map and map-model FSC curves, and local resolution estimates of the State 2 ElpABC-(HdrABC)$_2$ complex. **a & b**, State 2 ElpABC mobile-arm-focused map at 2.2 Å (a), and of Hdr(ABC)$_2$ at 2.1 Å (b). **c**, The consensus map at 2.3 Å obtained by using a mask including the ElpABC mobile arm and the Hdr regions. **d & e**, Local resolution maps obtained from the ElpABC mobile-arm-focused map (d) and the Hdr(ABC)$_2$ focused map (e), displayed for clarity over the State 2 composite map (grey, see Extended Data Fig. 4 and 5a). **f**, Map vs. model FSC curves of the masked and unmasked State 2 Elp-Hdr model (0.5 threshold, black line). **g**, Orientation distribution maps of each focused 3D-refinement.

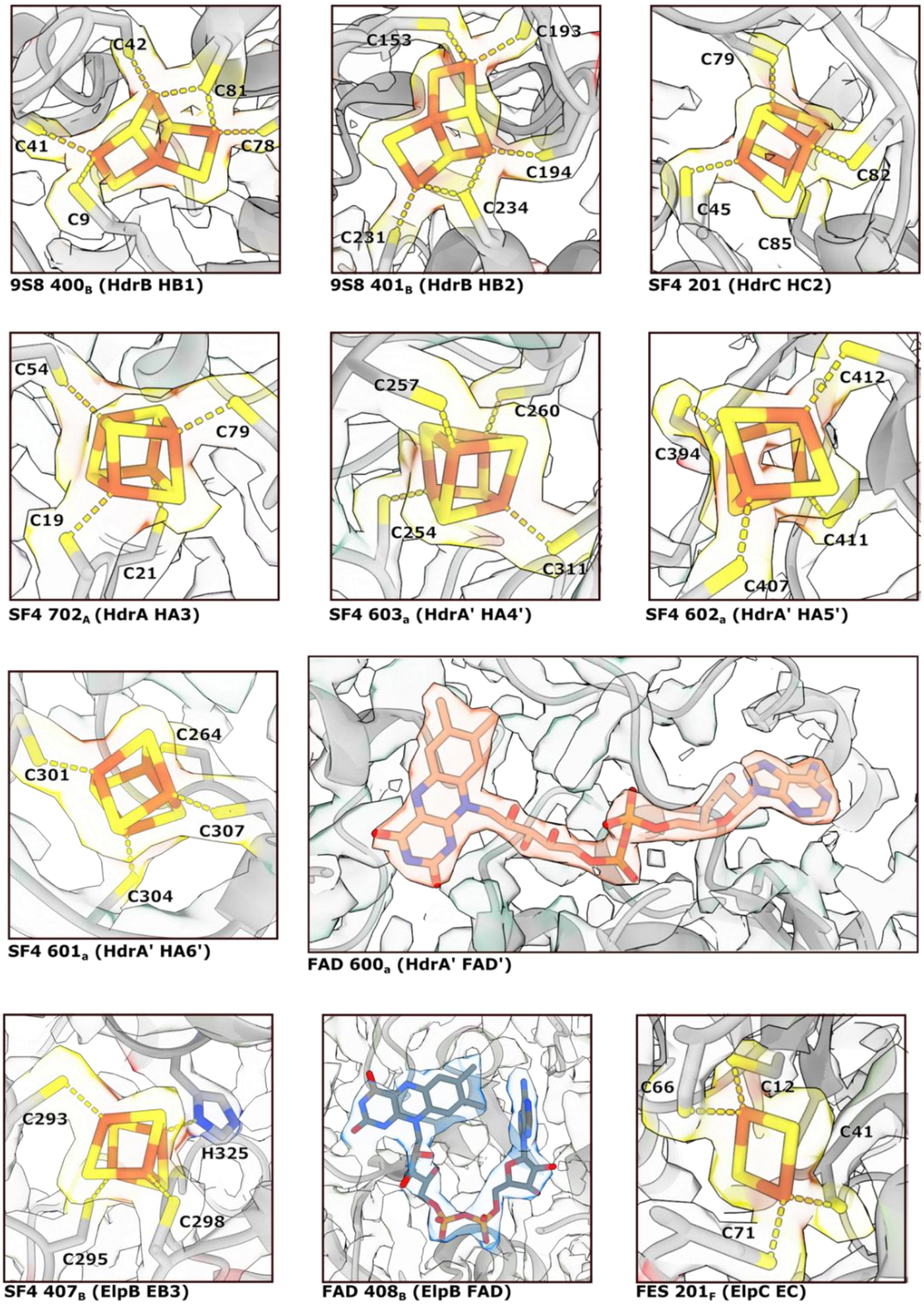

Supplementary Fig. 5 | Densities and models of the non-cubane iron-sulfur clusters of ElpB HB1 and HB2, the cubane iron-sulfur cluster of HdrC HC2, the cubane iron-sulfur cluster of HdrA HA3, three cubane iron-sulfur clusters of HdrA' (HA4', HA'5 and HA6'), the bifurcating FAD of HdrA' (FAD'), the His325-coordinated cubane iron-sulfur cluster of ElpB (EB3), the FAD of ElpB, and the ElpC 2Fe-2S cluster (EC).

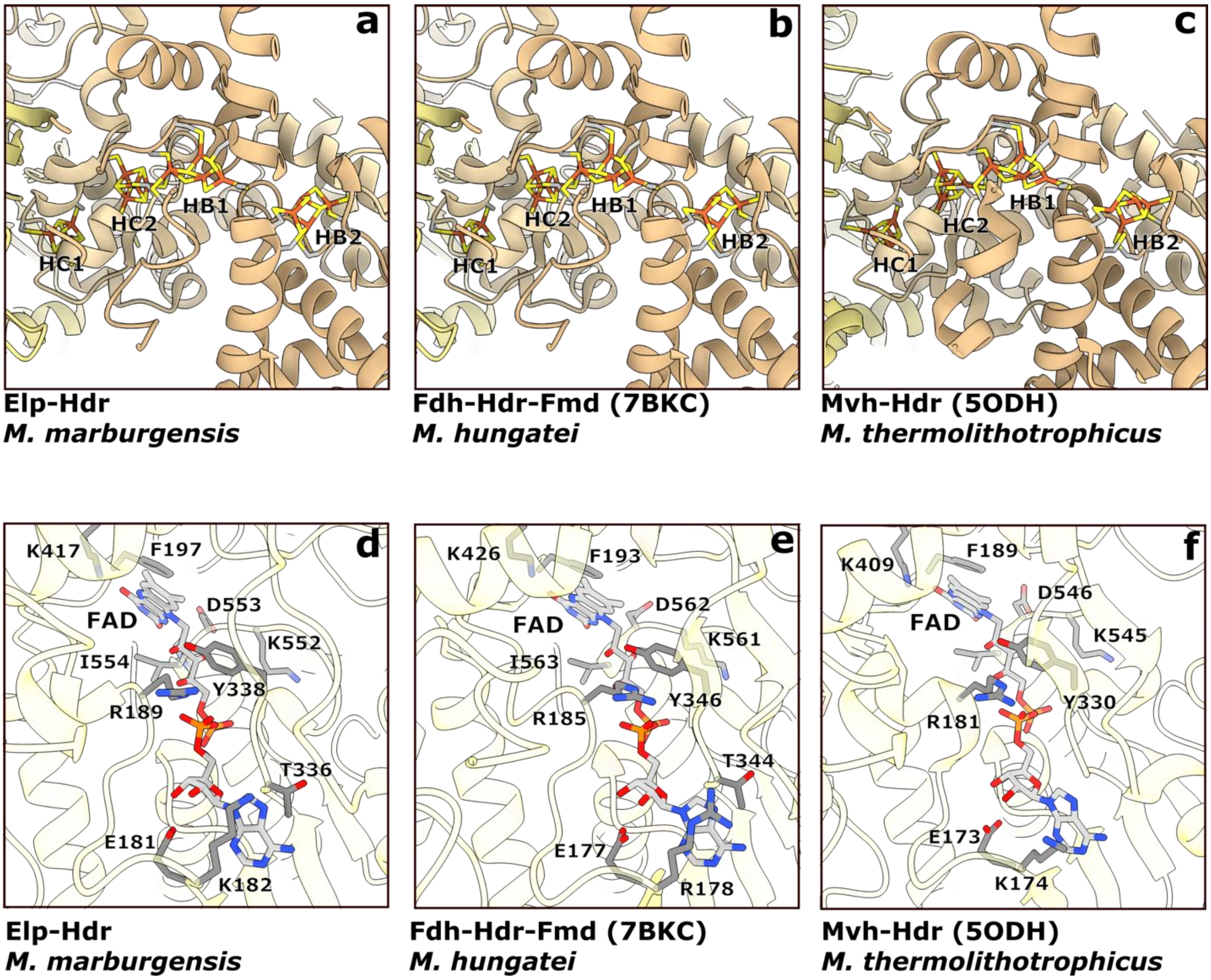

Supplementary Fig. 6 | The site of CoB-S-S-CoM reduction in HdrB (a-c) and the FAD-binding site (d-f) displays a very similar conformation in Elp-Hdr (**a**), Fdh-Hdr-Fmd in *M. hungatei* (PDB:7BKC) (**b**) and Mvh-Hdr of *M. thermolithotrophicus* (PDB:5ODH) (**c**). The cubane iron-sulfur clusters of HdrC (HC1 and HC2), the non-cubane iron-sulfur clusters of HdrB (HB1 and HB2), and the HdrA-bound FAD (FAD) and the amino acids involved in FAD binding are shown as sticks.

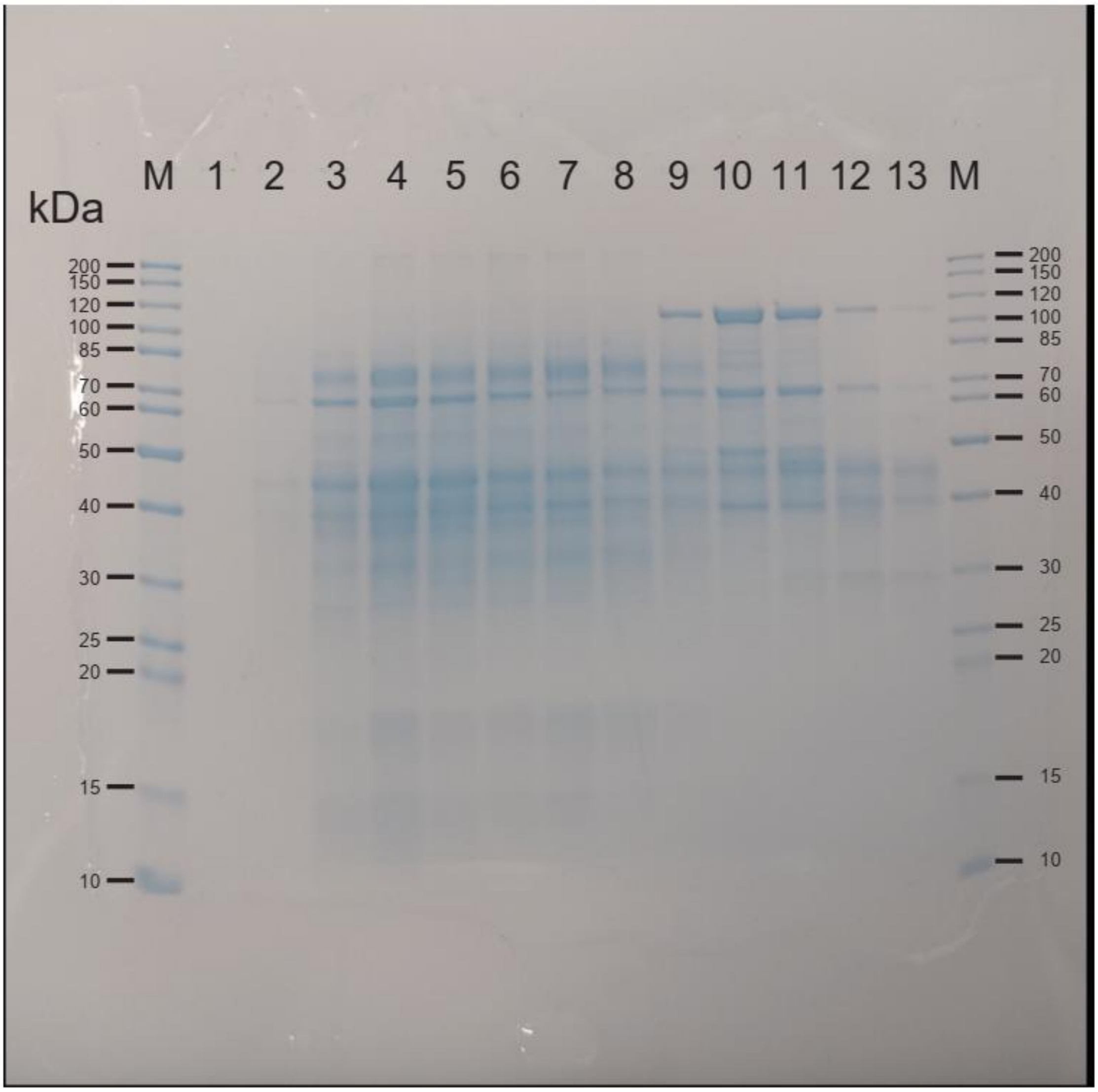

Supplementary Fig. 7 | Raw and uncropped data for gels used for Extended Data Fig. 2.

Supplementary Table. 1 | Presence or absence of the genes encoding Hmd, ElpAB, FdhAB, and MvhD homologs. Only representative methanogens are listed. The presence or absence of the genes encoding the respective proteins is indicated by a plus (or the protein annotation number with amino acid length, AA) or minus sign.

		Hmd	ElpA<br>ElpA-like	ElpB	FdhA	FdhB	ElpC<br>MvhD
Class I	***Methanobacteriales***						
	Methanobacterium aggregans DSM 29498	-	-	-	683 AA<br>WP_209584296.1	398 AA<br>WP_209584298.1	+
	Methanobacterium arcticum DSM 19844	+	376 AA<br>WP_048082405.1	382 AA<br>WP_048082404.1	682 AA<br>WP_211251468.1	400 AA<br>WP_048082953.1	+
	Methanobacterium bryantii DSM 863	+	368 AA<br>WP_069585784.1	382 AA<br>WP_069585785.1	684 AA<br>WP_069584796.1	424 AA<br>WP_083241031.1	+
	Methanobacterium formicicum DSM 3637	+	365 AA<br>EKF85605.1	382 AA<br>WP_004030869.1	683 AA<br>WP_004029641.1	398 AA<br>WP_004029640.1	+
	Methanobacterium petrolearium DSM 22353	+	366 AA<br>WP_209625833.1	382 AA<br>WP_209625834.1	-	-	+
	Methanobrevibacter boviskoreani DSM 25824	+	343 AA<br>WP_040681868.1	385 AA<br>WP_040681869.1	688 AA<br>WP_040682316.1	411 AA<br>WP_040682317.1	+
	Methanobrevibacter millerae DSM 16643	+	342 AA<br>WP_149732530.1	381 AA<br>WP_188118153.1	721 AA<br>WP_149731764.1	399 AA<br>WP_149731763.1	+
	Methanobrevibacter olleyae DSM 16632	+	359 AA<br>WP_067147079.1<br>360 AA<br>WP_074798894.1	393 AA<br>AMK15662.1<br>385 AA<br>WP_074798895.1	683 AA<br>WP_067145132.1	402 AA<br>WP_067145134.1	+
	Methanobrevibacter smithii DSM 861	+	369 AA<br>WP_337745186.1	382 AA<br>WP_244371170.1	702 AA<br>WP_244371713.1	401 AA<br>WP_278738707.1	+
	Methanobrevibacter woesei DSM 11979	+	-	-	685 AA<br>WP_116670212.1	403 AA<br>WP_116670211.1	+
	Methanothermobacter marburgensis DSM 2133	+	342 AA<br>WP_013296313.1	382 AA<br>WP_202965421.1	887 AA<br>WP_013294976.1	-	+
	Methanothermobacter thermautotrophicus DSM 3720	+	347 AA<br>WP_074359411.1	380 AA<br>WP_074359410.1	-	-	+
	Methanothermobacter wolfeii DSM 2970	+	347 AA<br>WP_283169005.1	380 AA<br>WP_283169004.1	681 AA<br>NLM03269.1	395 AA<br>NLM03270.1	+
	Methanothermus fervidus	+	340 AA<br>ADP77525.1	380 AA<br>ADP77524.1	685 AA<br>ADP77493.1	392 AA<br>ADP77494.1	+
	Methanococcales						
	Methanocaldococcus bathoardescens	+	377 AA<br>WP_048201331.1	379 AA<br>WP_173400797.1	676 AA<br>WP_081874445.1	-	+
	Methanocaldococcus fervens	+	-	-	678 AA<br>WP_015791209.1	379 AA<br>WP_015791210.1	+
	Methanocaldococcus jannaschii	+	378 AA<br>WP_010869499.1	379 AA<br>WP_010869498.1	673 AA<br>WP_083774551.1	-	+
	Methanocaldococcus lauensis	+	377 AA<br>CAB3287815.1	379 AA<br>CAB3287817.1	674 AA<br>WP_214400216.1	-	+
	Methanocaldococcus vulcanius	+	378 AA<br>WP_048196765.1	379 AA<br>WP_015732788.1	677 AA<br>WP_015733241.1	-	+
	Methanococcus aeolicus DSM 17508	+	-	-	677 AA<br>WP_011973295.1	374 AA<br>WP_011973294.1	+
	Methanococcus maripaludis DSM 2067	+	-	-	674 AA<br>WP_104838308.1	387 AA<br>WP_104838309.1	+
	Methanococcus voltae DSM 1537	+	-	-	677 AA<br>WP_209590740.1	377 AA<br>WP_209590741.1	+
	Methanothermococcus okinawensis	+	373 AA<br>WP_048057819.1	380 AA<br>WP_013866368.1	677 AA<br>WP_083810053.1	379 AA<br>WP_013867375.1	+
	Methanothermococcus thermolithotrophicus DSM 2095	+	384 AA<br>WP_018153231.1	392 AA<br>WP_018153230.1	675 AA<br>WP_083876336.1	384 AA<br>WP_018154419.1	+
	Methanotorris formicicus DSM 16983	+	374 AA<br>WP_007044706.1	379 AA<br>WP_007044707.1	688 AA<br>WP_083820854.1	379 AA<br>WP_007044685.1	+
	Methanotorris igneus DSM 5666	+	398 AA<br>WP_013799329.1	379 AA<br>WP_013799328.1	692 AA<br>WP_013798911.1	-	+
	Methanopyrales						
	Methanopyrus kandleri AV19	+	-	-	685 AA<br>WP_394296044.1	377 AA<br>WP_011018692.1	+

	Methanopyrus sp. KOL6	+	-	-	678 AA WP_232452976.1	387 AA WP_088336286.1	+
	Methanopyrus sp. SNP6	+	-	-	678 AA WP_236953835.1	387 AA WP_148689254.1	+
Class II	***Methanosarcinales***						
	Methanolobus bombayensis	-	-	-	-	-	-
	Methanolobus profundi	-	482 AA WP_091935829.1 *1	383 AA WP_091935830.1	-	-	+ *2
	Methanolobus psychrophilus	-	488 AA AFV25143.1*1	383 AA AFV25142.1	-	-	+
	Methanolobus tindarius	-	485 AA WP_023843923.1 *1	383 AA WP_023843924.1	-	-	+ *2
	Methanolobus vulcani	-	-	-	-	-	-
	Methanosalsum natronophilum	-	-	-	689 AA WP_259134760.1	384 AA WP_259134758.1	+ *3
	Methanosarcina acetivorans	-	-	-	-	-	+ *3
	Methanosarcina barkeri MS	-	-	-	703 AA AKB55894.1	401 AA AKB55893.1	+ *3
	Methanosarcina horonobensis	-	523 AA WP_048139202.1 *1	409 AA WP_048139203.1	-	-	+
	Methanosarcina soligelidi	-	531 AA WP_048051415.1 *1	409 AA WP_048051416.1	-	-	+
	Methanimicrococcus blatticola	-	563 AA WP_133516714.1 *1	425 AA WP_133516715.1	-	-	+
	Methanocellales						
	Methanocella arvoryzae	-	-	-	703 AA WP_012035111.1 685 AA WP_012034604.1	369 AA WP_012035112.1 414 AA WP_012034603.1	+
	Methanocella conradii	-	-	-	699 AA WP_014406694.1	367 AA WP_014406693.1	+ *2
	Methanocella paludicola	-	-	-	686 AA WP_012900321.1 706 AA WP_012901153.1	413 AA WP_012900320.1 370 AA WP_012901152.1	+ *2
	Methanomicrobiales						
	Methanocorpusculum parvum	-	390 AA WP_095641887.1 *4	412 AA WP_095641886.1	689 AA WP_095642451.1	414 AA WP_095642450.1	+
	Methanocorpusculum bavaricum	-	390 AA WP_042699327.1 *4	412 AA WP_042699330.1	689 AA WP_042698504.1	414 AA WP_042698507.1	+
	Methanocorpusculum labreanum	+	390 AA WP_011833429.1*4	412 AA WP_011833430.1	689 AA WP_011832838.1	414 AA WP_011832837.1	+
	Methanogenium organophilum	-	526 AA WP_268187233.1 *1	412 AA WP_268187234.1	688 AA WP_268186843.1	411 AA WP_268186844.1	+
	Methanolacinia paynteri	+	519 AA WP_048152740.1 *1	414 AA WP_048152741.1	684 AA WP_048152750.1 688 AA WP_048152943.1	414 AA WP_048152751.1 418 AA WP_048152942.1	+
	Methanolacinia petrolearia	+	519 AA WP_013328993.1 *1	414 AA WP_013328992.1	688 AA WP_013329561.1 684 AA WP_013328981.1	418 AA WP_013329560.1 414 AA WP_013328980.1	+
	Methanomicrobium mobile	-	525 AA WP_042706631.1 *1	414 AA WP_042706630.1	689 AA WP_042706614.1	414 AA WP_042706613.1	+
	Methanoregula boonei	-	-	-	621 AA WP_012106466.1	417 AA WP_012106467.1	+
	Methanoregula formicica	+	-	-	689 AA WP_015284671.1 686 AA WP_015284701.1	383 AA WP_015284672.1 417 AA WP_015284702.1	+
	Methanospirillum hungatei	-	-	-	687 AA WP_011448817.1 688 AA WP_011448797.1	425 AA WP_011448816.1 414 AA WP_011448798.1	+
	Archaeoglobales						
	Ca. Methanoglobus hypatiae	-	-	-	-	-	+

	Archaeoglobus fulgidus DSM 8774	-	-	-	-	-	+
	Archaeoglobus neptunius	-	-	-	-	-	+
	Archaeoglobus profundus DSM 5631	-	-	-	956 AA WP_012940074.1	185 AA WP_012940075.1	+
	Archaeoglobus veneficus DSM 11195	-	-	-	957 AA WP_013683412.1	184 AA WP_013683411.1	+
**Thermoproteota phylum**	**Nitrososphaeria (formerly Thaumarchaeota)**						
	Thaumarchaeota JZ-2 bin_220 (described as methanogenic)	-	-	-	-	-	+
	**Methanomethylicia (formerly Verstraetearchaeota)**						
	Ca. Methanosuratincola subterraneus	-	-	-	-	-	+
	Ca. Methanosuratus petracarbonis	-	-	-	-	-	+
	Ca. Methanomethylicus oleusabulum	-	-	-	-	-	+
	Ca. Methanomethylicus mesodigestum Nezhaarchaeales WYZ-LMO7 and WYZ-LMO8 (described as methanogenic)	-	-	-	-	-	+
	Korarchaeia						
	Ca. Methanodesulfokores washburnensis	-	-	-	-	-	+

*[1] Mostly, ElpA-like (~500 amino acids) is longer than ElpA (~350-370 amino acids) and the predicted structure is similar to FdhA rather than EplA (see Extended Data Fig. 8a).
*[2] Have both HdrA fused MvhD and MvhD.

*[3] Have only HdrA fused MvhD.

*[4] The length of ElpA-like of *Methanocorpusculum* sp. is 390 amino acids and its predicted structure is similar to ElpA.

*5 *Archaeoglobus profundus* and *A. veneficus* contain bacterial Fdh likely obtained via lateral gene transfer.
