# Supplementary material for: Electron flow in hydrogenotrophic methanogens under nickel limitation
Source: Nature. 2025 Jul 2;644(8076):490–6. doi: 10.1038/s41586-025-09229-y (PMC12350162; doi:10.1038/s41586-025-09229-y)
Supplement: Supplementary file 2 — Reporting Summary [file 41586_2025_9229_MOESM2_ESM.pdf]

nature portfolio

Corresponding author(s): Seigo Shima and Bonnie J. Murphy

Last updated by author(s): 2025-05-14

Reporting Summary

Nature Portfolio wishes to improve the reproducibility of the work that we publish. This form provides structure for consistency and transparency in reporting. For further information on Nature Portfolio policies, see our Editorial Policies and the Editorial Policy Checklist.

Statistics

For all statistical analyses, confirm that the following items are present in the figure legend, table legend, main text, or Methods section.

n/a	Confirmed	
☐	☒	The exact sample size (*n*) for each experimental group/condition, given as a discrete number and unit of measurement
☐	☒	A statement on whether measurements were taken from distinct samples or whether the same sample was measured repeatedly
☒	☐	The statistical test(s) used AND whether they are one- or two-sided<br>*Only common tests should be described solely by name; describe more complex techniques in the Methods section.*
☒	☐	A description of all covariates tested
☒	☐	A description of any assumptions or corrections, such as tests of normality and adjustment for multiple comparisons
☐	☒	A full description of the statistical parameters including central tendency (e.g. means) or other basic estimates (e.g. regression coefficient) AND variation (e.g. standard deviation) or associated estimates of uncertainty (e.g. confidence intervals)
☒	☐	For null hypothesis testing, the test statistic (e.g. *F*, *t*, *r*) with confidence intervals, effect sizes, degrees of freedom and *P* value noted<br>*Give P values as exact values whenever suitable.*
☒	☐	For Bayesian analysis, information on the choice of priors and Markov chain Monte Carlo settings
☒	☐	For hierarchical and complex designs, identification of the appropriate level for tests and full reporting of outcomes
☒	☐	Estimates of effect sizes (e.g. Cohen's *d*, Pearson's *r*), indicating how they were calculated

Our web collection on statistics for biologists contains articles on many of the points above.

Software and code

Policy information about availability of computer code

Data collection	EPU (Thermo Fisher Scientific)
Data analysis	SEQUEST HT embedded in Proteome Discoverer 1.4, DIA-NN 1.8, CryoSPARC Live, cryoSPARC v3.2 and 4.1; Relion 4.0; Relion 5.0; CTFFind4.1; Pyem; Phenix 4487; Coot 0.9; ChimeraX; Q-Score; Model Angelo

For manuscripts utilizing custom algorithms or software that are central to the research but not yet described in published literature, software must be made available to editors and reviewers. We strongly encourage code deposition in a community repository (e.g. GitHub). See the Nature Portfolio guidelines for submitting code & software for further information.

Data

Policy information about availability of data

All manuscripts must include a data availability statement. This statement should provide the following information, where applicable:

- Accession codes, unique identifiers, or web links for publicly available datasets
- A description of any restrictions on data availability
- For clinical datasets or third party data, please ensure that the statement adheres to our policy

The primary data supporting all reported results within this paper are available from the corresponding authors on reasonable request. The proteome raw data were deposited in Pride. State 1 Elp-Hdr model was deposited in the Protein Data Bank (PDB) with the accession code 8RVY and the composite map in the Electron Microscopy Data Bank (EMBD) with the code 19538. This composite map has the associated focused maps with the codes EMD-19536 (State 1 mobile-arm-focused map), EMD-19535 (State 1 Hdr-focused map) and the consensus map EMD-19537 (State 1 Elp-Hdr consensus map). State 2 Elp-Hdr asymmetric model was [illegible]

deposited in the PDB as 8RVU, and the composite map as 19533; the State 2 dimer model was deposited in the PDB as 8RVV, and the dimer composite map as EMD-19534. These composite maps have the associated focused maps with the codes EMD-19531 (State 2 mobile-arm-focused map), EMD-19530 (State 2 Hdr-focused map) and the consensus map EMD-19532 (State 2 Elp-Hdr consensus map). Finally, the Hdr(ABC)2 model was deposited in the PDB as 8RWN and the map in the EMDB as EMD-19564. The sequences used for the prediction were obtained from the UniProtKB database: M. marburgensis HdrA (Q50756), MvhB (P60232) and FmdF (D9PU52). The sequences used for the prediction were obtained from the UniProtKB database: M. marburgensis HdrA (Q50756), MvhB (P60232) and FmdF (D9PU52). The mass spectrometry proteomics data have been deposited to the ProteomeXchange Consortium via the PRIDE partner repository.

Research involving human participants, their data, or biological material

Policy information about studies with human participants or human data. See also policy information about sex, gender (identity/presentation), and sexual orientation and race, ethnicity and racism.

Reporting on sex and gender	This paper does not involve human participants or their data.
Reporting on race, ethnicity, or other socially relevant groupings	This paper does not involve human participants or their data.
Population characteristics	This paper does not involve human participants or their data.
Recruitment	This paper does not involve human participants or their data.
Ethics oversight	This paper does not involve human participants or their data.

Note that full information on the approval of the study protocol must also be provided in the manuscript.

Field-specific reporting

Please select the one below that is the best fit for your research. If you are not sure, read the appropriate sections before making your selection.

☒ Life sciences ☐ Behavioural & social sciences ☐ Ecological, evolutionary & environmental sciences

For a reference copy of the document with all sections, see nature.com/documents/nr-reporting-summary-flat.pdf

Life sciences study design

All studies must disclose on these points even when the disclosure is negative.

Sample size	For structural data, the number of micrographs collected was determined by available microscope time. The sample size of kinetic and proteome studies are described in the figures and supplementary source files.
Data exclusions	Particle images were selected for further analysis by visual inspection of 2D and 3D class features, according to established practice in the field. No other data were excluded. No data were excluded in the experiments of kinetics and proteomic analysis.
Replication	Described in the figures.
Randomization	For all image analysis and reconstruction, particles were automatically assigned to random data half-sets by the refinement software. Randomization is not applicable for the microbiological, kinetic, and proteomic studies.
Blinding	We got the data from the cells of an isolated methanogenic archaeon; therefore, blinding is not applicable for this work.

Reporting for specific materials, systems and methods

We require information from authors about some types of materials, experimental systems and methods used in many studies. Here, indicate whether each material, system or method listed is relevant to your study. If you are not sure if a list item applies to your research, read the appropriate section before selecting a response.

Materials & experimental systems

n/a	Involved in the study	
☒	☐	Antibodies
☒	☐	Eukaryotic cell lines
☒	☐	Palaeontology and archaeology
☒	☐	Animals and other organisms
☒	☐	Clinical data
☒	☐	Dual use research of concern
☒	☐	Plants

Methods

n/a	Involved in the study	
☒	☐	ChIP-seq
☒	☐	Flow cytometry
☒	☐	MRI-based neuroimaging

Plants

Seed stocks	We do not use plants in this work.
